# Immune response and reactogenicity after immunization with two-doses of an experimental COVID-19 vaccine (CVnCOV) followed by a third-fourth shot with a standard mRNA vaccine (BNT162b2): RescueVacs multicenter cohort study

**DOI:** 10.1016/j.eclinm.2022.101542

**Published:** 2022-07-01

**Authors:** Ana Ascaso-del-Rio, Javier García-Pérez, Mayte Pérez-Olmeda, Eunate Arana-Arri, Itziar Vergara, Carla Pérez-Ingidua, Mercedes Bermejo, María Castillo de la Osa, Natale Imaz-Ayo, Ioana Riaño Fernández, Oliver Astasio González, Francisco Díez-Fuertes, Susana Meijide, Julio Arrizabalaga, Lourdes Hernández Gutiérrez, Humberto Erick de la Torre-Tarazona, Alberto Mariano Lázaro, Emilio Vargas-Castrillón, José Alcamí, Antonio Portolés, RescueVac study Group

**Affiliations:** a Clinical Pharmacology Department, Hospital Clínico San Carlos, IdISSC, C/ Prof Martín Lagos s/n, 28040 Madrid, Spain; b AIDS Immunopathogenesis Unit, Instituto de Salud Carlos III (ISCIII), Cra Pozuelo km2, Majadahonda, 28220 Madrid, Spain; c Centro de Investigación Biomédica en Red de Enfermedades Infecciosas (CIBERINFEC), Instituto de Salud Carlos III (ISCIII), Madrid, Spain; d Serology Laboratory, Centro Nacional de Microbiologia, Instituto de Salud Carlos III (ISCIII), Madrid, Spain; e Biocruces Bizkaia Health Research Institute, Barakaldo, Spain; f Primary Care Research Group, Biodonostia Health Research Institute, San Sebastian, Spain; g Donostia University Hospital, Biodonostia Health Research Institute, San Sebastián, Spain; h Servicio de Medicina Preventiva, Hospital Clínico San Carlos, IdISSC, Madrid, Spain; i Pharmacology and Toxicology Department, School of Medicine, Universidad Complutense de Madrid (UCM), Madrid, Spain; j Instituto de Investigación Sanitaria del hospital Clínico San Carlos

**Keywords:** COVID-19 vaccines, mRNA vaccines, Heterologous vaccination, BNT162b2, CVnCoV, Fourth dose, neutralizing antibodies, Boost doses of vaccines, Omicron, Experimental vaccines, Antibodies, Immune response

## Abstract

**Background:**

There is no evidence to date on immunogenic response among individuals who participated in clinical trials of COVID-19 experimental vaccines redirected to standard national vaccination regimens.

**Methods:**

This multicentre, prospective controlled cohort study included subjects who received a COVID-19 experimental vaccine (CVnCoV)(test group, TG) - and unvaccinated subjects (control group, CG), selected among individuals to be vaccinated according to the Spanish vaccination program. All study subjects received BNT162b2 as a standard national vaccination schedule, except 8 (from CG) who received mRNA-1273 and were excluded from immunogenicity analyses. Anti-RBD antibodies level and neutralising titres (NT50) against G614, Beta, Mu, Delta and Omicron variants were analysed. Reactogenicity was also assessed.

**Findings:**

130 participants (TG:92; CG:38) completed standard vaccination. In TG, median (IQR) of anti-RBD antibodies after first BNT162b2 dose were 10740·0 BAU/mL (4466·0-12500) compared to 29·8 BAU/mL (14·5-47·8) in CG (*p* <0·0001). Median NT50 (IQR) of G614 was 2674·0 (1865·0-3997·0) in TG and 63·0 (16·0-123·1) in CG (*p* <0·0001). After second BNT162b2 dose, anti-RBD levels increased to ≥12500 BAU/mL (11625·0-12500) in TG compared to 1859·0 BAU/mL (915·4-3820·0) in CG (*p* <0·0001). NT50 was 2626·5 (1756·0-5472·0) and 850·4 (525·1-1608·0), respectively (*p* <0·0001). Variant-specific (Beta, Mu, Omicron) response was also assessed. Most frequent adverse reactions were headache, myalgia, and local pain. No severe AEs were reported.

**Interpretation:**

Heterologous BNT162b2 as third and fourth doses in previously suboptimal immunized individuals elicit stronger immune response than that obtained with two doses of BNT162b2. This apparent benefit was also observed in variant-specific response. No safety concerns arose.

**Funding:**

Partly funded by the Institute of Health Carlos-III and COVID-19 Fund, co-financed by the European Regional Development Fund (FEDER) “A way to make Europe”.

**Research in context**

**Evidence before this study**

Reports on immune response elicited by either homologous or heterologous vaccination regimes against SARS-CoV-2 are becoming increasingly demanded day to day. Moreover, evidence on extra and booster doses in individuals primed according to their national vaccination programs is lately growing. However, immune response and dynamics of those subjects who were primed with experimental vaccines eventually not authorised before receiving an authorised vaccine included in national programs is unknown.

**Added value of this study**

As to our knowledge, this is the first report of immune response elicited after receiving 4 doses of heterologous COVID-19 vaccines in general population, and neutralizing activity against variants of concern including Omicron.

**Implications of all the available evidence**

Periodical boosters of heterologous COVID-19 vaccines, even including priming with a suboptimal vaccine, elicit more potent immune response than two dose-based schemes, which bring us to hypothesise that response induced after mixing and matching authorised vaccines could be even stronger. These results also point to thousands of individuals participating in experimental vaccines trials resulting unsuccessful may experience a benefit in their immunity when re-vaccinated under national programs. Furthermore, although our findings suggest a poor neutralization of Omicron variant, subjects administered three or four doses of heterologous vaccines maintained significantly higher neutralising antibody titres than those receiving a standard two doses course.

Alt-text: Unlabelled box

## Introduction

Since the first SARS-CoV-2 variants of concern were described in September 2020, numerous new variants have emerged worldwide.^1^ As a consequence, national vaccination plans and new vaccine developments are being challenged at short intervals. On the one hand, the immunogenicity and efficacy of first-generation vaccines must be revised and assessed for new variants, in particular those considered as variants of concern (VoC) – Beta (B.1.351), Gamma (P.1), Delta (B.1.617.2), and recently Omicron (B.1.1.529) in Europe as of January 2022.^1^ Results have shown substantial reduction of overall efficacy with some variants,^2^ which has led public health authorities to revise initial one or two dose-based vaccination plans to consider a third dose, especially in groups at highest risk of severe COVID-19. In fact, more than 15% population in the European Union has received an additional dose as of December 2021,^3^ since the European Medicines Agency (EMA) issued recommendations on extra doses and boosters of mRNA-based vaccines.^4^ However, the potential need of fourth and further doses, the population to whom should be administered, and the interval to set between doses are hot matters of debate. On the other hand, there are currently numerous vaccine candidates at different stages of development,^5^ some of them will succeed while others will not. It is worth mentioning that subjects participating in trials of candidates eventually unsuccessful need to be re-directed to receive full vaccination according to national plans. These individuals not only will have received 3 or 4 doses of vaccines but a heterologous regime to adequately complete their vaccination. Currently there is an increasing body of evidence supporting both heterologous regimes and immunization with a third dose of authorised vaccines.^6^, ^7^, ^8^, ^9^ However less is known regarding heterologous vaccination including prematurely stopped candidates, or more than 3 doses of COVID vaccines. One of these candidates was the mRNA-based CVnCoV SARS-CoV-2 vaccine (CureVac AG). CVnCoV is a chemically unmodified mRNA vaccine candidate encoding a stabilised, full-length, native SARS-CoV-2 spike protein that is delivered by lipid nanoparticles. In a phase 1 dose-escalation study, two doses of CVnCoV administered 28 days apart were safe and immunogenic inducing both antibodies targeting the spike of SARS-CoV-2 and neutralising antibodies.^10^ Based on these results a phase 2b/3 clinical trial including more than 37.000 volunteers from ten countries of Europe and Latin America was started. Given the mean overall efficacy against symptomatic COVID-19 of 48·2% recently reported,^11^ CureVac announced to cancel further development of this vaccine in favour of a second generation one (https://www.ema.europa.eu/en/documents/withdrawal-letter/withdrawal-letter-curevacs-covid-19-vaccine-cvncov_.pdf). Near 20,000 individuals – more than 5,000 of them in Europe – received at least one dose in the abovementioned study and had to be re-vaccinated according to applicable national plans. It is yet unknown how strong the immune response will be in them and whether it will be similar or not to that elicited after a standard immunization course.

Here, we present results of the RescueVac study, a controlled cohort study to describe humoral immunogenicity and reactogenicity after standard vaccination in cohorts of subjects with or without previous administration of the experimental CVnCoV SARS-CoV-2 vaccine.

## Methods

### Study design and population

This study was designed as a prospective controlled cohort study to assess immunogenicity and reactogenicity after immunization with a third and a fourth dose of a standard mRNA COVID-19 vaccine in a population already primed with a suboptimal experimental vaccine before recruitment.

The enrollable individuals were those planned to be vaccinated according to the national COVID-19 vaccination program in three Spanish centres (San Carlos University Hospital –Madrid–, Cruces University Hospital –Baracaldo–, Donostia University Hospital –San Sebastián–) from 14th July to 15th September 2021. In the absence of previous applicable data, sample size was estimated as a pilot study approach. It was expected to include at least 90 participants (ratio 2:1 -test:reference- for a more reliable estimation of the group with higher induction effects expected), enough for the estimation in previous serologic COVID-19 studies. Among them, there could be subjects who had not received any COVID-19 vaccine before and subjects having previously received an unauthorised COVID-19 vaccine (CVnCoV) as participants in clinical trials from development programs. Although there were no protocol restrictions in experimental vaccines which participants may have been primed with, only individuals treated with CVnCoV vaccine coincided with the present study time schedule. Regarding vaccines from the national program, BNT162b2 (Tozinameran: Comirnaty, BioNTech, Germany) was the vaccine largely administered at these sites. This study made no intervention on the immunoprophylaxis to be received by the participants.

Immunocompromised or patients with clinically significant unstable medical condition were excluded from the study, as the focus was on providing information applicable to the general population. No age limit was established, nor was time limit since prime vaccination. Subjects were informed about the study in the vaccination point of each study site. All volunteers signed informed consents to donate their samples and to participate in the study before inclusion. Samples from individuals who had already received the experimental CVnCoV vaccine were included in the test group for immunogenic analyses, while those from subjects who had not previously received any vaccine against SARS-CoV-2 formed the control group. All of them were immunized with two doses of BNT162b2.

This study was reviewed and approved by the Ethics Committee of the San Carlos University Hospital (21/528-E) and complied with ethical principles of the Declaration of Helsinki and Good Clinical Practice, as well as the applicable Spanish law. The study is registered with the Spanish Register of Clinical Studies (REEC–code: 0061-2021-OBS).

### Assessments

Response to vaccination was assessed as per a) levels of antibodies to the SARS-CoV-2 spike protein receptor binding domain (S-RBD) and b) neutralizing antibodies titres before first and second vaccine doses, and at 2–5 weeks after full vaccination. Neutralization of Beta, Delta, Mu, and Omicron SARS-CoV-2 variants was specifically studied. Immunogenicity data were collected before the first (baseline, V1) and second dose (V2) of the vaccine from the national program, and at 2–5 weeks after full vaccination (V3). Reactogenicity, i.e. adverse reactions reported within 10 days after each BNT162b2 dose, was also assessed at V1 and V2. Safety was assessed throughout the study. Data collection was fulfilled by the same physicians along the study and following the same procedures regardless of the study group.

#### Anti-RBD antibody immunoassay

Antigen-specific humoral immune response was analysed using Elecsys Anti-SARS-CoV-2 S assay (Roche Diagnostics, Mannheim, Germany) an electrochemiluminescence immunoassay used to detect antibodies (including IgG) to the SARS-CoV-2 S-RBD on the Cobas e411 module (Roche Diagnostics, Mannheim, Germany). According to the manufacturer, the measuring range spanned 0·4 U/mL to 250 U/mL (up to 2500 U/mL with onboard 1:10 dilution, and up to 12500 U/mL with onboard 1:50 dilution). Values higher than 0·8 BAU/mL were considered positive. Correlation between U/ml and BAU (International OMS standard) is U=0.972 BAU.

#### Virus neutralization assays

To measure neutralising antibody titres, diluted plasma samples were preincubated with pseudoviruses carrying optimized sequences for the expression of spike variants 614G (B.1), Beta (B.1.351) Delta (B.1.617.2), Mu (B.1.621) and Omicron (B.1.1.529). Pseudoviral particles were generated by cotransfection of the plasmid pNL4-3ΔenvRen and expression vectors for the different viral spike variants cloned in the pcDNA3·1-SCoV2Δ19 plasmid and added at a concentration of 10 ng p24Gag per well to Vero E6 cells in 96-well plates. At 48 h post infection, viral infectivity was assessed by measuring luciferase activity (Renilla Luciferase Assay, Promega, Madison, WI, USA) using a 96-well plate luminometer LB 960 Centro XS³ (Berthold Technologies, Oak Ridge, TN, USA). The titre of neutralising antibodies was calculated as 50% inhibitory dose (neutralising titre 50, NT50), expressed as the reciprocal of four-fold serial dilution of heat-inactivated sera (range 1:32–1:131·072), resulting in a 50% reduction of pseudovirus infection compared with control. Samples below the detection threshold (1:32 serum dilution) were given 1:16 value. Non-specific neutralisation was assessed using a related pseudovirus expressing the vesicular stomatitis virus envelope.

### Statistical analysis

A descriptive analysis of demographic and clinical variables, days elapsed between visits and vaccinations, and antibody levels was performed. Data were summarised using absolute and relative frequencies for categorical variables and mean, range and SD or median and IQR for numerical variables. For immunogenicity statistics, geometric means were calculated and reported together with their 95% confidence interval (CI). The proportion of participants with serum conversion was also computed. With regard to safety and reactogenicity, adverse events (AEs) were coded using the current version of the Medical Dictionary for Regulatory Activities (MedDRA) and reported until the database lock date of Dec 7, 2021. Continuous variables were tested for normality hypothesis assumption.

The inferential analysis was conducted to identify any significant changes in variables over time and between groups. Log transformation was calculated for antibodies results. As many test group participants reached the highest measurable antibody titre, lognormal transformation of some data failed to result in a symmetric distribution, so t-tests or non-parametric Wilcoxon signed-rank test and Mann-Whitney tests were conducted to compare quantitative data over time and between groups, respectively. Geometric means ratio (GMR) were calculated and reported. All tests were reported two-sided maintaining a 95% CI. The α error for statistical significance in primary analysis was corrected to 0·025 as per Bonferroni to adjust for multiple comparisons. Missing data were not imputed.

The primary analysis compared the anti-RBD increase achieved at visits 2 and 3 with respect to baseline, between groups. Secondary analysis for immunogenicity outcomes was performed on neutralizing antibodies increase achieved at visits 2 and 3 with respect to baseline, between groups; on antibody levels and neutralizing titres reached at each visit, on the intragroup differences in titres over the time and on the days elapsed between visits and between vaccination regimens and doses. These analyses were performed on a modified intention-to-treat basis (mITT) excluding participants with previous confirmed SARS-CoV-2 infection and control cases who were seropositive at baseline. Besides this, during the descriptive phase of the analysis, it was detected that only 8 subjects, none of them from the test group, had received a vaccine other than BNT162b2, namely mRNA-1273 (Spikevax, Moderna Biotech). Considering this, it was decided to exclude these 8 subjects from immunogenicity analysis to avoid possible bias in the interpretation of results. The decision was made *a priori* and a sensitivity analysis was subsequently performed including these subjects. Additional immunogenicity analyses by subgroups were also conducted, and adjusted by: diagnosis of COVID-19, baseline seropositivity, and concomitant immunomodulators. The analysis population for reactogenicity and safety included all participants who received a vaccine.

Statistical analysis was carried out using statistical software SPSS® release 26 (IBM Corp., Armonk, NY, USA) and Stata® release 16·1 (StataCorp LLC, College Station, TX, USA).

### Role of the funding source

Funders had no role in study design, data analysis, interpretation, writing of the report or decision to submit. ISCIII grants supported personnel hiring and reagents used in serology assays. AA, JGP and CPI, had access and revised dataset; EAA, IV, JA and APP took the decision for publication.

## Results

**Figure 1:**
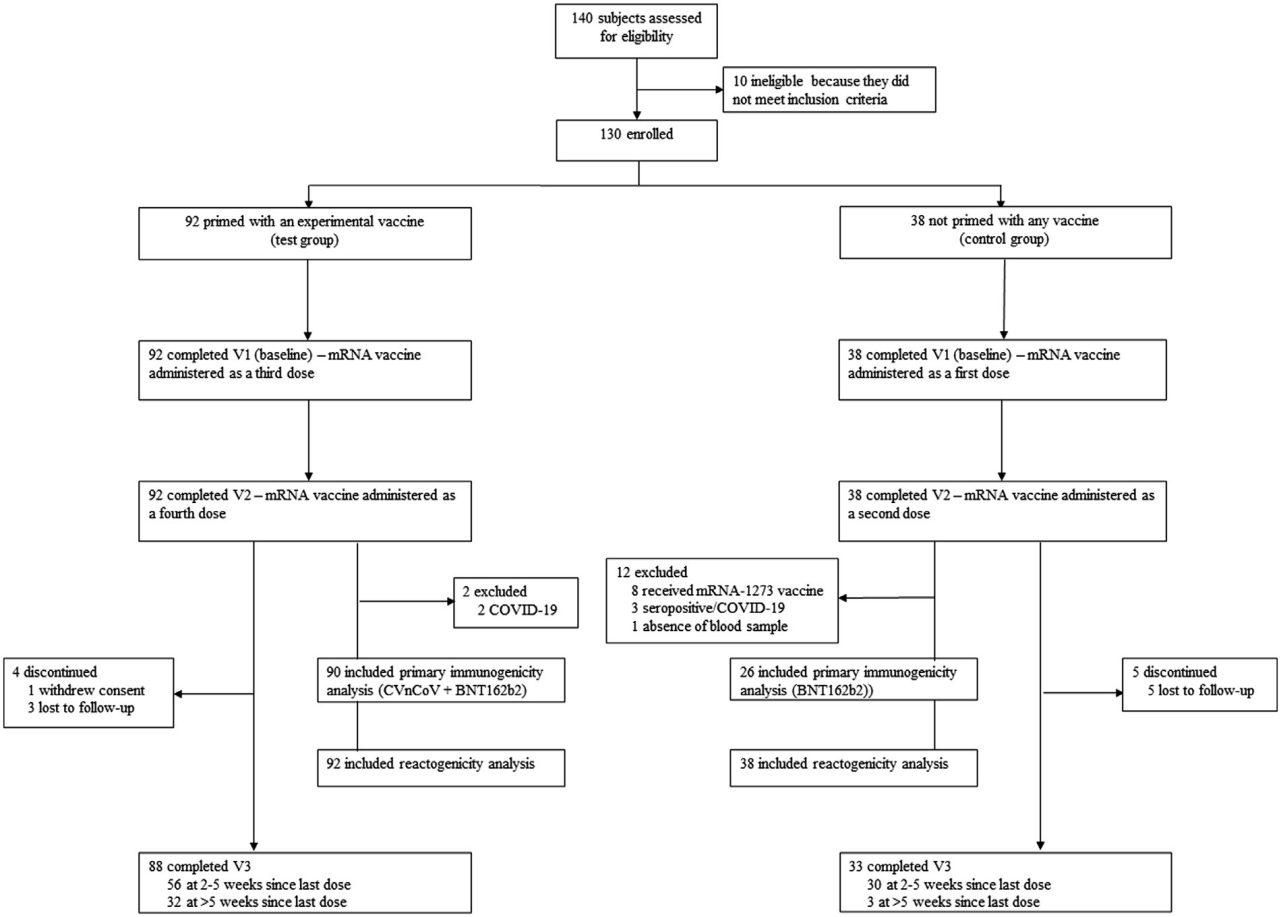
Study profile.

Between July 14th and August 8th, 2021, 140 subjects were enrolled into the study, of whom 10 were excluded before the first dose – non-compliant with selection criteria – and 9 discontinued between second and third visit. In total, 130 participants (92 in the test group and 38 in the control group) completed vaccination during the study and had first and second blood tests drawn, and 121 (n= 88 and n= 33, respectively) completed visit 3 (Figure 1). As mentioned above, 8 of these control group subjects were excluded from immunogenicity analyses as they received mRNA-1273 vaccine instead of BNT162b2 (appendix 1 p 2).

**Figure 2:**
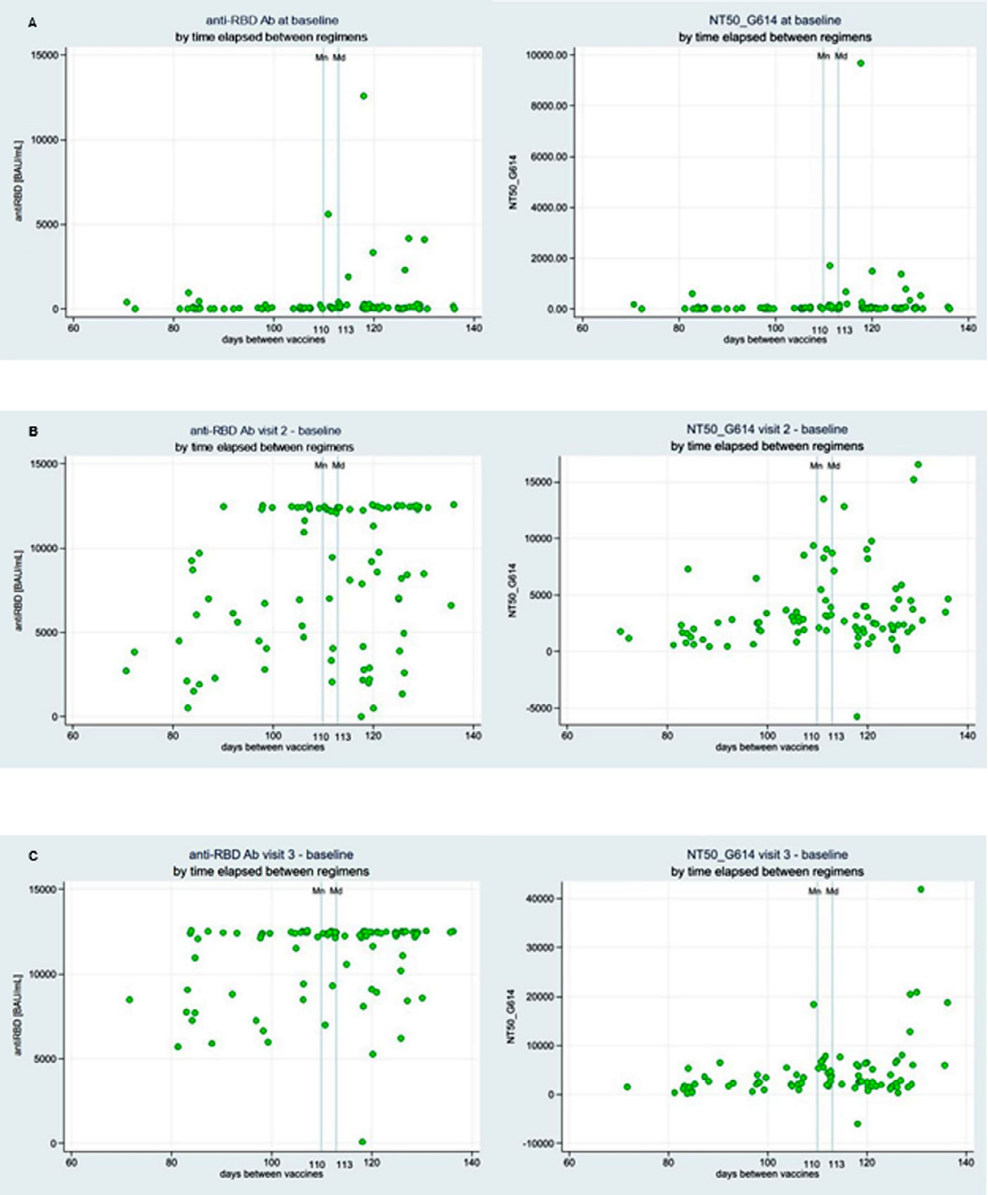
**Anti-RBD levels and G614 NT50 in the test group a) at baseline (before first dose of BTN162b2), b) from baseline to first dose of BTN162b2, and c) from baseline to second dose of BTN162b2, related to days elapsed since last dose of CVnCoV**. Each green dot represents a subject.

Baseline characteristics were not matched but demographics were similar between groups (Table 1 and appendix 1 p 3). Median age of participants was 26 (IQR 24-28) and 27 (IQR 23-45) years in control and test group respectively; 33% and 39%, respectively, were female. In the test group, mean time elapsed between the last dose of the first vaccination course and first dose of the standard course (third jab) was 110 days (SD 15·4). No significant correlation was observed between this interval and antibody levels at baseline (Figure 2). Mean (SD) interval between BNT162b2 doses was 21 days (2·3) and 21 days (0·9) in the test group and control group, respectively. It should be noted that 35 subjects made visit 3 later than 35 days since last dose (mean 28 days [SD 14·0] in the test group and 20 days [9·2] in the control group]). There were 4 cases of confirmed COVID-19 infection (all of them with mild symptoms): two of them (control group) took place several months before the study and the remaining two (test group) occurred between 6 and 8 days after the first dose of BNT162b2. In the test group, one participant was on biological treatment for atopic dermatitis with topical tacrolimus and topical corticosteroids, another one received Hepatitis A and HPV vaccines between visit 2 and 3 and one more received Vivotif® (Typ21a vaccine) after the second dose of BNT162b2.

**Table 1:** Baseline characteristics; mITT population.

	Control group (n=30) (BNT162b2)	Test group (n= 92) (CVnCoV + BNT162b2)	Overall (n=122)
**Age**			
Mean (SD)	28 (8·7)	32 (12·0)	31 (11·4)
Median (IQR)	26 (24–28)	27 (23–45)	27 (23–39)
**Sex, n (%)**			
Female	10 (33%)	36 (39%)	46 (38%)
Male	20 (67%)	56 (61%)	76 (62%)
**Interval between CVnCoV (2^nd^ dose) and BNT162b2 (3^rd^ dose) (days)**			
Mean (SD)	NA	110 (15·4)	110 (15·4)
Median (IQR)	NA	113 (99-122)	113 (99-122)
**Interval between BNT162b2 doses (days)**			
Mean (SD)	21 (0·9)	21 (2·3)	21 (2·0)
Median (IQR)	21 (21-21)	21 (21-21)	21 (21-21)
**Seropositivity, n (%)**			
Anti-RBD levels	0 (0)	89 (98·9%)	89 (73·0%)
Neutralising titres	0 (0)	28 (31·1%)	28 (23·0%)
**Confirmed COVID-19, n (%)**			
>6 months before inclusion	2 (6·7%)	0 (0)	2 (1·6%)
Peri-vaccination	0 (0)	2 (2·2%)	2 (1·6%)
**Concomitant immunomodulators, n (%)**			
Topical tacrolimus/corticosteroids	0 (0)	1 (1·1%)	1 (0·8%)
Other vaccines (HAV, HPV, Ty21a)	0 (0)	2 (2·2%)	2 (1·6%)
**Comorbidities, n (%)**			
Cancer	0 (0)	0 (0)	0 (0)
Immune deficiencies	0 (0)	0 (0)	0 (0)
Solid organ/stem cell transplant	0 (0)	0 (0)	0 (0)
Chronic kidney disease	0 (0)	0 (0)	0 (0)
Liver disease	0 (0)	0 (0)	0 (0)
Chronic pulmonary disease	0 (0)	0 (0)	0 (0)
Asthma	1 (3·3%)	4 (4·3%)	5 (4·1%)
Hypertension	2 (6·7%)	2 (2·2%)	4 (3·3%)
Heart disease	0 (0)	2 (2·2%)	2 (1·7%)
Diabetes	0 (0)	0 (0)	0 (0)
Neurological disease	0 (0)	0 (0)	0 (0)
Depression	1 (3·3%)	3 (3·3%)	4 (3·3%)
Thalassaemia	0 (0)	0 (0)	0 (0)
Atopic dermatitis/allergic disease	1 (3·3%)	4 (4·3%)	5 (4·1%)
Pregnancy	0 (0)	0 (0)	0 (0)

mITT: modified intention-to-treat.

**Figure 3:**
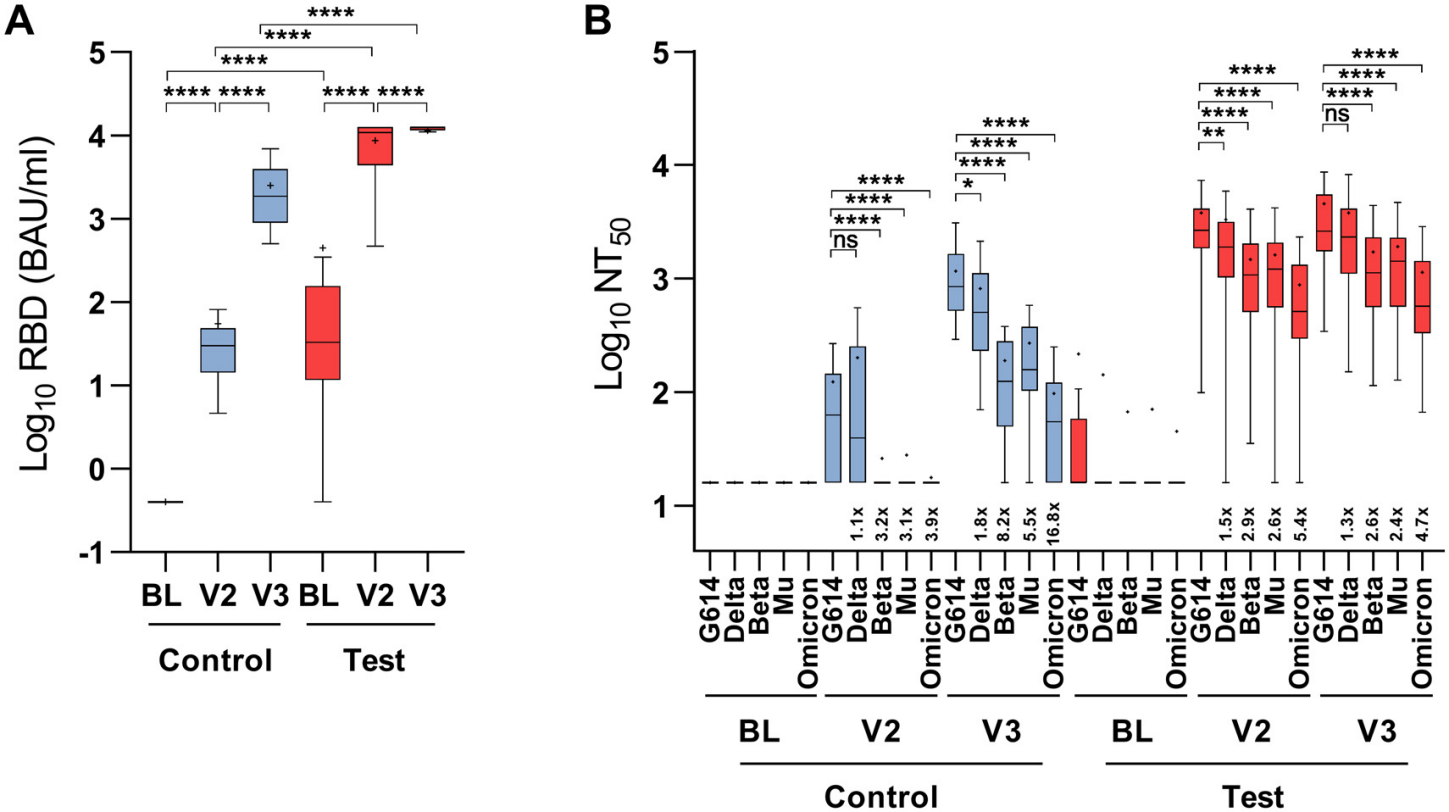
**Evolution of a) anti RBD-antibodies concentration (ECLIA) and b) neutralizing titres against reference G614, Delta (B.1.617.2), Beta (B.1.351), Mu (B.1.621) and Omicron (B.1.1.529) variants, by study group**. Dashes inside boxes indicated the median value and crosses indicated the arithmetic mean. Box limits indicate the interquartile range (IQR). Whiskers are adjusted to maximal and minimal values if lower than 1.5 times the IQR. BL: baseline (before first dose of BTN162b2). V2: 3 weeks after fist BTN162b2 dose. V3: 4 weeks after second dose of BTN162b2. *p <0·05. **p <0·005. ****p <0·0001. ns: non-significant. *p*-values obtained using unpaired two-tailed nonparametric Mann-Whitney test.

With regard to immunogenicity, concentration of RBD and neutralizing antibodies at each visit are shown in Table 2 and Figure 3, and in appendix 1 pp 4-5. First dose of BNT162b2 induced stronger humoral immune response in the test group as compared to the control group. Median (IQR) levels of anti-RBD antibodies were 10740·0 BAU/mL (4466·0-12500) and 29·8 BAU/mL (14·5-47·8), respectively (*p* <0·0001), and geometric mean levels (GMT) were 7199·0 BAU/mL (95% CI 6189·0-8373·8) and 29·8 BAU/mL (95% CI 19·0-46·7), respectively. Regarding neutralising antibody titres, median (IQR) of G614 variant NT50 was 2674·0 (1865·0-3997·0) in the test group and 63·0 (16·0-123·1) in the control group (*p* <0·0001); GMT were 2659·8 (95% CI 2204·7-3209·0) and 65·6 (95% CI 41·4-103·9), respectively. BNT162b2 booster further increased anti-RBD antibody titre in the test group that remained significantly higher than levels achieved in the control group (median ≥12500 BAU/mL [IQR 11625·0-12500] vs 1859·0 BAU/mL [915·4-3820·0]), *p* <0·0001; GMT 11181·0 [95% CI 10658·2-11730·0] vs 1832·7 [95% CI 1262·6-2660·4]). NT50 were barely and not significantly increased in the test group after BNT162b2 booster but remain significantly higher than in the control group at visit 3 (median 2626·5 [IQR 1756·0-5472·0] vs 850·4 [525·1-1608·0], *p* <0·0001; GMT 2990·5 [95% CI 2471·8-3618·0] vs 956·4 [95% CI 712·2-1284·4]) (Figure 3, Tables 2 and 3). The anti-RBD GMR between tests and controls ranged from 241·8 (95% CI 168·1-347·8) at visit 2 (21 days after first BNT162b2 dose) to 6·1 (95% CI 5·0-7·5) at visit 3 (5 weeks after second BNT162b2 dose) (Table 2), even though no participants from the control group achieved the highest measurable titre of anti-RBD antibodies (12500 BAU/mL) whereas in the test group 1 participant did at V1, 38 (42·4%) at V2, and 63 (70·9%) at V3. Accordingly, a three-fold increase in GM of neutralising titres against the reference G614 (B1.1) variant was found after full immunization with BTN162b2 in the CVnCoV group in comparison with control subjects (Table 2).

**Table 2:** RBD antibody levels and neutralization titres at each visit; mITT population.

	Control group (n=27) (BNT162b2)				Test group (n= 92) (CVnCoV & BNT162b2)				
	n	Mean (SD)	Median (IQR)	GMT (95% CI)	n	Mean (SD)	Median (IQR)	GMT (95% CI)	GMR (95% CI)
**SARS-CoV-2 anti-RBD (BAU/mL)**									
Baseline	27	0·4 (0)	0·4 (0·4-0·4)	0.4 (0·4-0·4)	92	444·0 (1567·7)	32·9 (11·7-151·4)	39·4 (25·6-60·7)	98·6 (44·4-218·8)
Visit 2	26	55·2 (74·1)	29·8 (14·5-47·8)	29·8 (19·0-46·7)	90	8720·5 (4213·1)	10740·0 (4466·0-12500)	7199.0 (6189·0-8373·8)	241·8 (168·1-347·8)
Visit 3	22	2517·2 (1985·8)	1859·0 (915·4-3820·0)	1832·7 (1262·6-2660·4)	86	11422·6 (2065·13)	12500 (11625·0-12500)	11181·0 (10658·2-11729·5)	6·1 (5·0-7·5)
**NT50 _ G614**									
Baseline	27	16·0 (0)	16·0 (16·0-16·0)	16·0 (16·0-16·0)	92	218·0 (1037·00)	16·0 (16·0-56·81)	33·2 (24·9-44·3)	2·1 (1·2-3·5)
Visit 2	26	123·1 (154·9)	62·96 (16·0-123·1)	65·6 (41·4-103·9)	90	3794·6 (3409·1)	2674·0 (1865·0-3997·0)	2659·8 (2204·7-3209·0)	40·5 (26·6-61·8)
Visit 3	22	1170·9 (754·8)	850·35 (525·10-1608·0)	956·4 (712·2-1284·4)	86	4593·9 (5828·6)	2626·5 (1756·0-5472·0)	2990·5 (2471·8-3618·0)	3·1 (2·1-4·6)
**NT50 _ Delta**									
Baseline	27	16·0 (0)	16·0 (16·0-16·0)	16·0 (16·0-16·0)	92	142·3 (622·90)	16·0 (16·0-16·0)	27·0 (20·9-34·8)	1·7 (1·1-2·7)
Visit 2	26	201·8 (358·9)	39·6 (16·0-252·1)	59·6 (32·1-110·7)	90	3325·1 (4558·0)	1910·0 (1035·0-3142·0)	1813·2 (1422·1-2312·0)	30·4 (17·5-52·8)
Visit 3	22	819·3 (920·7)	506·75 (235·5-1081·0)	517·4 (333·8-802·0)	86	3792·7 (4782·8)	2322·5 (1113·0-4073·0)	2276·9 (1827·0-2837·5)	4·4 (2·7–7·1)
**NT50 _ Beta**									
Baseline	27	16·0 (0)	16·0 (16·0-16·0)	16·0 (16·0-16·0)	92	67·1 (232·95)	16·0 (16·0-16·0)	21·7 (17·8-26·5)	1·4 (0·9-2·0)
Visit 2	26	26·1 (26·1)	16·0 (16·0-16·0)	20·4 (16·0-26·0)	90	1481·5 (1415·5)	1085·0 (525·1-2034·0)	926·0 (738·4-1161·3)	45·4 (29·2-70·4)
Visit 3	22	190·3 (196·1)	124·65 (50·3-272·3)	116·5 (72·7-186·5)	86	1720·0 (1642·7)	1130·5 (566·8-2300·0)	1158·8 (950·1-1413·3)	9·9 (6·4-15·8)
**NT50 _ Mu**									
Baseline	27	16·0 (0)	16·0 (16·0-16·0)	16·0 (16·0-16·0)	92	70·6 (310·7)	16·0 (16·0-16·0)	22·1 (18·2-26·8)	1·4 (1·0-2·0)
Visit 2	26	28·1 (30·3)	16·0 (16·0-16·0)	21·3 (16·5-27·4)	90	1621·8 (1549·5)	1221·5 (567·1-2066·0)	1042·6 (832·7-1305·3)	48·9 (31·7-76·0)
Visit 3	22	269·85 (282·6)	157·55 (108·7-369·2)	175·4 (113·1-271·8)	86	1911·8 (1821·2)	1422·5 (574·8-2267·0)	1268·3 (1035·5-1553·5)	7·2 (4·6-11·3)
**NT50 _ Omicron**									
Baseline	27	16·0 (0)	16·0 (16·0-16·0)	16·0 (16·0-16·0)	92	45·4 (179·00)	16·0 (16·0-16·0)	19·9 (17·0-23·3)	1·2 (0·9-1·7)
Visit 2	26	17·8 (9·2)	16·0 (16·0-16·0)	16·9 (15·1-18·9)	90	879·4 (968·9)	513·2 (297·0-1314·0)	493·5 (382·1-637·4)	29·2 (17·9-47·6)
Visit 3	22	97·8 (107·2)	54·8 (16·0-119·1)	57·0 (35·3-92·1)	86	1133·9 (1393·3)	574·55 (340·3-1424·0)	631·2 (496·9-801·9)	11·1 (6·6-18·6)

mITT: modified intention-to-treat; SD: standard deviation; IQR: interquartile range; GMT: geometric mean; IC: confidence interval; GMR: geometric mean ratio; NT50: 50% neutralising antibody titre.

**Table 3:** Changes in anti-RBD antibodies concentrations and NT50 response between visits (intra-group) and study groups (inter-group); mITT population.

	Control group (n=27) (BNT162b2)				Test group (n= 90) (CVnCoV & BNT162b2)				Inter-group comparisons		
		Absolute intragroup change				Absolute intragroup change					
	n	Mean (SD)	Median (IQR)	ZW	n	Mean (SD)	Median (IQR)	ZW	WW	Z	p -value
**SARS-CoV-2 anti-RBD (BAU/mL)**											
Visit 2 - Visit 1	26	54·8 (74·1)	29·4 (14·1-47·4)	4·46 (*p* <0·00001)	90	≥ 8267·3 (4202·0)	8911·1 (≥ 4235·9-12413·4)	8·24 (*p* <0·00001)	377	-7·57	<0·00001
Visit 3 - Visit 1	22	2516·8 (1985·8)	1858·6 (915·0-38919·6)	4·11 (*p*<0·00001)	86	≥ 10953·3 (2444·3)	12317·4 (≥ 9374·0-12465·5)	8·05 (*p* <0·00001)	287	-6·96	<0·00001
Visit 3 - Visit 2	21	2405·2 (1969·6)	1643·9 (890·6-3566·9)	4·02 (*p* <0·00001)	86	≥ 2642·8 (3301·4)	347 (≥ 0-4766·9)	6·26 (*p* <0·00001)	1309	1·41	<0·1615
**NT50 _ G614**											
Visit 2 - Visit 1	26	107·1 (154·9)	47·0 (0-107·1)	4·15 (*p*<0·00001)	90	3572·1 (3452·7)	2567 (1755·4-3981·0)	7·94 (*p*<0·00001)	392	-7·48	<0·00001
Visit 3 - Visit 1	22	1154·9 (75438)	834·4 (509·1-1592·0)	4·11 (*p*<0·00001)	86	4363·7 (5911·7)	2523·5 (1737·0-5456·0)	7·75 (*p*<0·00001)	554	-4·92	<0·00001
Visit 3 - Visit 2	21	1025·7 (706·7)	810·6 (469·1-1510·1)	4·02 (*p*<0·00001)	86	745·8 (5175·4)	34·0 (-704·0-1280·0)	0·80 (*p*=0·4282)	1497	2·85	0·0039
**NT50 _ Delta**											
Visit 2 - Visit 1	26	185·8 (358·9)	23·6 (0-236·1)	3·66 (*p*=0·0001)	90	3180·0 (4523·6)	1822·0 (996·0-2966·0)	8·24 (*p*<0·00001)	474	-6·94	<0·00001
Visit 3 - Visit 1	22	803·3 (920·7)	490·8 (219·5-1065·0)	4·11 (*p*<0·00001)	86	3643·8 (4743·9)	2236·2 (1089·0-3950·0)	8·05 (*p*<0·00001)	541	-5·02	<0·00001
Visit 3 - Visit 2	21	586·9 (930·1)	364 (151·0-1192·4)	3·77 (*p*=0·0001)	86	410·6 (5243·0)	233·5 (-686·0-880·0)	1·38 (*p*=0·1704)	1263	1·01	0·3159
**NT50 _ Beta**											
Visit 2 - Visit 1	26	10·1 (26·1)	0 (0-0)	2·00 (*p*=0·1250)	90	1413·3 (1389·5)	1069·0 (456·5-2003·3)	8·06 (*p*<0·00001)	387	-7·53	<0·00001
Visit 3 - Visit 1	22	174·3 (196·1)	108·7 (34·3-256·3)	4·06 (*p*<0·00001)	86	1649·4 (1642·6)	1114·5 (548·8-2223·5)	7·88 (*p*<0·00001)	367	-6·35	<0·00001
Visit 3 - Visit 2	21	157·9 (200·5)	105·8 (32·6-149·5)	3·83 (*p*<0·00001)	86	230·9 (1426·1)	28·6 (-455·0-511·2)	0·93 (*p*=0·3552)	1239	0·82	0·4152
**NT50 _ Mu**											
Visit2 - Visit 1	26	12·1 (30·3)	0 (0-0)	2·23 (*p*=0·0625)	90	1550·0 (1514·5)	1165·5 (551·1-1979·0)	8·24 (*p*<0·00001)	374·5	-7·62	<0·00001
Visit 3 - visit 1	22	253·9 (282·6)	141·6 (92·7-353·2)	4·09 (*p*<0·00001)	86	1837·4 (1821·5)	1387·0 (535·4-2251·0)	7·93 (*p*<0·00001)	409	-6·03	<0·00001
Visit 3 - visit 2	21	234·9 (291·21)	128·2 (73·59-267·1)	3·93 (*p*<0·00001)	86	278·24 (1365·87)	166·7 (-338·8-542·0)	1·96 (*p*=0·0497)	1187	0·42	0·6826
**NT50 _ Omicron**											
Visit2 - Visit 1	26	1·8 (9·2)	0 (0-0)	1·0 (*p*=0·3173)	90	833·3 (955·8)	489·1 (270·9-1238·0)	8·11 (*p*<0·00001)	393	-7·21	<0·00001
Visit 3 - Visit 1	22	81·8 (107·2)	38·8 (0-103·1)	3·72 (*p*=0·0001)	86	1086·4 (1394·1)	554·25 (289·4-1408·0)	7·86 (*p*<0·00001)	398	-6·11	<0·00001
Visit 3 - Visit 2	21	82·5 (111·4)	37·7 (0-99·7)	3·59 (*p*=0·0001)	86	233·5 (846·0)	63·26 (-163·0-323·9)	2·12 (*p*=0·0337)	1079	0·07	0·9442

mITT: modified intention-to-treat; SD: standard deviation; IQR: interquartile range; IC: confidence interval; NT50: 50% neutralising antibody titre; WMW = Mann Whitney U test. WW= Wilcoxon W test. ZW= Wilcoxon signed-rank test.

Importantly, although 98·9% of participants previously immunized with CVnCoV were seropositive at baseline, anti-RBD levels and NT50 were detected at low levels (median 32·88 BAU/mL [IQR 11.68-151.36] and 16·0 [16·0-56.8]), respectively; anti-RBD GMT 39·4 BAU/mL [95% CI 25·6-60·7] and NT50 GMT 19·9-33·2 [17.0-44.3], respectively). As expected, baseline seropositive participants had higher response compared with seronegative participants in both post-dose visits (appendix 1 pp 6). In addition, increase of anti-RBD levels and NT50 in the test group with respect to the interval between CVnCoV and BTN162b2 vaccines is represented in Figure 2b and 2c.

Sensitivity analyses performed after removal of 35 participants exceeding 5 weeks (35 days) for visit after second BTN162b2 dose (visit 3) (appendix 1 pp 7-9) and 7 subjects with potential confounding factors (i.e. confirmed COVID-19 infection, immunosuppressant (analysis not shown) did not reveal significant changes in anti-RBD levels and NT50 results.

Neutralizing activity against pseudoviruses carrying the spike protein from SARS-CoV-2 variants was compared between both groups (Figure 3 and Table 3). Similarly, as for the reference G614 variant neutralization titres were significantly higher against Beta, Delta, Mu, and Omicron variants in the test group. Unexpectedly neutralization against variants with strong resistance to neutralization dropped significantly less in subjects previously immunized with CVnCoV as compared to controls. Indeed, in the control group after two doses of BTN162b2, geometric mean of NT50 was 8·2, 5·5 and 16·8 folds lower against Beta, Mu, and Omicron variants, respectively, in comparison with NT50 against reference G614 virus. In contrast, in subjects previously immunized with CVnCoV such difference was 2·6, 2·4 and 4·7 folds against Beta, Mu, and Omicron variants. Furthermore, neutralizing potency against Beta and Mu was above 1:1000 NT50 and above 1:500 against Omicron in the test group, which is about tenfold that found in the control group (appendix 1 p 10).

Regarding reactogenicity, local and systemic reactions observed within 10 days after each BNT162b2 dose were similar between groups, being headache and myalgia the most common systemic reactions, and pain the most frequent local reaction (Table 4; appendix 1 pp 11-12). The intensity of the events was mild in 80.6% of the cases and moderate in 19.4%. No severe or serious adverse events were reported. No association factors between participants´ degree of reactogenicity and age or baseline status could be identified.

**Table 4:** Reactogenicity from 0 to 10 days after each BTN162b2 dose by study group.

	Control group (*n*=27) (BNT162b2)								Test group (*n*=92) (CVnCoV & BNT162b2)								mRNA-1273 (*n*=8)
	N° of adverse events						N° of subjects		N° of adverse events						N° of subjects		
	NAE	%	Mild	Moderate	Severe	SAE	n	%	NAE	%	Mild	Moderate	Severe	SAE	n	%	n
Injection site pain	13	20·97	10	3	0	0	9	33·33	47	26·86	34	13	0	0	35	38·04	0
Headache	15	24·1	13	2	0	0	8	29·63	34	19·43	28	6	0	0	22	23·91	0
Myalgia	6	9·68	3	3	0	0	4	14·81	13	7·43	7	6	0	0	11	11·96	0
Swelling at injection site	4	6·45	4	0	0	0	4	14·81	14	7·95	13	1	0	0	14	15·22	0
Fatigue	4	6·45	2	2	0	0	2	7·41	14	7·95	13	1	0	0	11	11·96	0
Injection site redness	2	3·23	2	0	0	0	2	7·41	11	6·29	10	1	0	0	10	10·87	0
Chills	1	1·61	1	1	0	0	1	3·70	9	5·14	9	0	0	0	8	8·70	0
Arthralgia	3	4·84	1	2	0	0	1	3·70	5	2·86	4	1	0	0	5	5·43	0
Stinging at injection	5	8·06	5	0	0	0	3	11·1	5	2·86	5	0	0	0	4	4·35	0
Discomfort	3	4·84	2	1	0	0	2	7·41	5	2·86	4	1	0	0	5	5·43	0
Arthromyalgia	1	1·61	1	0	0	0	1	3·70	6	3·43	6	0	0	0	5	5·43	0
Diarrhoea	1	1·61	1	0	0	0	1	3·70	2	1·14	2	0	0	0	2	2·17	0
Fever	0	0	0	0	0	0	0	0	3	1·71	2	0	0	0	2	2·17	0
Nasal congestion	0	0	0	0	0	0	0	0	2	1·14	0	2	0	0	1	1·09	0
Tiredness	0	0	0	0	0	0	0	0	2	1·14	1	1	0	0	2	2·17	0
Abnormal menstrual cycle	1	1·61	1	0	0	0	1	3·70	0	0	0	0	0	0	0	0·00	0
Itching at injection site	1	1·61	1	0	0	0	1	3·70	0	0	0	0	0	0	0	0·00	0
Axillary lymphadenopathy	1	1·61	1	0	0	0	1	3·70	1	0·57	1	0	0	0	1	1·09	0
Body pain	0	0	0	0	0	0	0	0	1	0·57	1	0	0	0	1	1·09	0
Lumbar discopathy	1	1·61	0	0	0	0	1	3·70	0	0	0	0	0	0	0	0·00	0
Sore throat	0	0	0	0	0	0	0	0	1	0·57	1	0	0	0	1	1·09	0
**Totals**	62	-	48	14	0	0	14	-	175	-	141	33	0	0	51	-	0

NAE = number of adverse events; *n* = number of subjects affected.

## Discussion

Our study aimed to obtain efficient and valuable information on multiple doses of vaccine, of great importance given the evolution of SARS-CoV-2 pandemics and increase of VoCs. As per the situation in the centres involved in the study, all the patients included in the test group had received CVnCoV – two doses –, a chemically unmodified mRNA vaccine^12^ in relatively low doses (12 μg, vs 30 μg for BNT162b2 and 100 μg for mRNA-1273). Despite promising immunogenicity results derived from phase 1,^10^ CVnCoV efficacy against COVID-19 resulted sub-optimal in later-phase clinical trials,^11^ arising some questions on immune status of participants redirected to standard vaccination programs. Our results show that individuals previously immunized with CVnCoV exhibited low but consistent levels of anti-RBD antibodies and neutralizing activity against SARS-CoV-2 after 2·3 to 4·5 months. Such low levels are not unexpected and could be a consequence of waning observed three months after immunization, as described for COVID-19 vaccines.^13^ Importantly, in these subjects primed with CVnCoV vaccine, subsequent two doses of a standard mRNA vaccine, in this case preferentially BNT162b2, could be considered as a third and fourth immunisation with a heterologous regimen. Actually, despite the persistence of immune memory, antibody decay increases the risk of SARS-CoV-2 infection and a third dose becomes necessary to achieve protection against asymptomatic and symptomatic infections, particularly in aged groups above 60 and patients with risk factors for developing severe COVID-19.^14^, ^15^, ^16^ In some settings administration of a fourth dose is currently under study. Taking this into consideration, our data show that the first dose of BNT162b2 in participants previously vaccinated with two doses of CVnCoV resulted in a booster response that elicited anti-RBD antibodies at a level 242-fold (GMT) higher than in the control group. After the second dose of BNT162b2 antibody response remained 6-fold (GMT) higher in the test group compared to controls. Actually, these differences are certainly higher than observed, as in the test group 70% of subjects reached the highest level of RBD that is measurable by the technique. Non-parametric statistical test used to assure the interpretation of results confirmed this in both cases as well as by the sensitivity analysis.

Heterologous immunization with a second dose of RNA results in better immunogenicity and antibody levels than homologous regimens with ChAdOx1 nCov-19.^7^^,^^17^ Similar results, have been described when a third dose with RNA is administered to subjects previously immunized with two doses of other vaccines.^18^ Interestingly heterologous immunization with a third dose results not only in higher antibody levels over homologous immunization but increases the efficacy against symptomatic disease (from 50% to 68%) at population level.^19^

A limitation of this study is the lack of a control group vaccinated with three doses of BNT162b. In the COV-Boost study^17^ a third dose with CVnCoV in patients previously vaccinated with BNT162b was not optimal and anti-spike IgG antibodies were three-fold higher in the group that received homologous (BNT162b2) or heterologous (mRNA-1273) booster suggesting that the order in the administration of the different vaccines can be important to obtain best results.

Neutralization data are particularly interesting not only because the high titres of neutralising antibodies elicited but because their activity against VoCs and VoIs. In the control group, after the second dose of BNT162b2 a sharp decrease of neutralising titres against Beta (8·2 fold), Mu (5·5 fold) and particularly Omicron (16·8 fold) was observed due to partial immune escape of these variants, as previously described.^20^^,^^21^ In contrast, in the test group, after a first dose of BNT162b2 not only high neutralization titres were obtained against all variants but a lower fold decrease – indicating better neutralization efficacy – was found against Beta, Mu, and Omicron (2·9, 2·6, and 5·4 fold, respectively) when compared with the reference G614 variant. A booster dose of BNT162b2 did not modify significantly neutralization activity in the test group. These results suggest that the combination of two CVnCoV doses and one BNT162b2 boost generates antibodies with higher affinity that effectively neutralize VoCs that escape from humoral immunity, as compared to two doses of BNT162b2. Our data suggest strong protection against VoCs including Omicron, as very recently it has been reported that vaccine recipients with post vaccination NT50 titres above 100 had estimated vaccine efficacy of 91% (87–94%).^22^ It has been suggested that both timing between prime and booster and between booster and a third dose of vaccines favours the frequency and maturation of B-memory lymphocytes and improve the generation of high-avidity antibodies by increased somatic mutation.^13^^,^^23^ In our study the time elapsed between CVnCoV and BNT162b2 booster probably plays a major role in the induction of potent neutralization responses observed. In this setting it is important to note that booster with BNT162b2 was efficient in the induction of RBD and neutralizing antibodies in the control group that were “naïve” for immunization as described in phase 3 clinical trials. However, this booster dose of BNT162b2 barely increased levels of NT50 titres in the test group that was previously primed, even at low efficacy, with the CVnCoV prototype. This finding supports the concept that a booster dose should not be given in few weeks after a second immunization or in people convalescent of natural infection; rather, a longer time should be considered to immunize with additional vaccine doses.

Finally, one major consequence deducible from our work is that immunization with CVnCoV generates durable immune memory through the induction of memory B-cells although the immune stimuli provided is unable to induce high levels of anti-SARS-CoV-2 antibodies. This finding is crucial for booster strategies to strengthen immune response against SARS-CoV-2 and increase efficacy against VoCs, particularly in people vaccinated with vaccines displaying lower efficacy and waning antibody response and considering the current approach aimed at repeating multiple booster doses. Concerning reactogenicity, the absence of severe events, a profile of reactions consistent with that already known, and a similar distribution of events between groups allow us to keep calm, to date, with regard to added doses of RNA vaccines.

One limitation of the study is that only subjects from the test group received a third dose of a COVID-19 vaccine, preventing us from further comparisons. Also, all subjects from the test group had participated only in CVnCoV clinical trials, which limits our understanding of immune response and dynamics after standard vaccine courses in individuals who participated in trials of other unauthorised vaccines. Finally, the observational nature of the study does not allow us to adequately control potential biases resulting from the lack of randomization and non-blinded allocation. Notwithstanding, in our opinion the consistency of our results with others on response after a third dose of a COVID-19 vaccine attenuates this limitation and add value to our findings after a fourth dose, given the scarcity of evidence on these matters available to date.

In conclusion, heterologous third and fourth doses with BNT162b2 in previously immunized CVnCoV people result in anti-RBD and neutralising titres higher than obtained with standard prime-boost regimen, with no concerns regarding reactogenicity. Further studies on immunogenicity and efficacy of booster and extra doses of COVID-19 vaccines are warranted to better understand immune response against SARS-CoV-2 infection and help to decision-making in public health plans.

## Contributors

AA: Methodology, Data curation, Formal analysis, Visualization, Writing – Original draft. JGP: Data curation, Validation, Investigation, Formal analysis, Visualization. MPO: Validation, Investigation. EAA: Investigation, Supervision. IV: Investigation, Supervision. CPI: Investigation, Data curation. MB: Investigation. MCO: Investigation. NIA: Investigation. IRF: Investigation. OAG: Investigation. FDF: Investigation, Formal analysis. SM: Investigation. JAr: Investigation. LGH: Investigation. ET: Investigation. AML: Investigation. EVC: Investigation. JA: Conceptualization, Methodology, Writing – Review & Editing, Supervision. AP: Conceptualization, Methodology, Writing – Review & Editing, Supervision.

## Data sharing statement

The study protocol is provided in the appendix 1 (pp 14 et seq.). Raw data are available upon requests directed to the corresponding author; after approval of a proposal, data can be shared through a secure online platform.

## Declaration of interests

Biocruces Bizkaia HRI and Curevac have a clinical trial contract unrelated to the present study. Biodonostia HRI and Curevac have a clinical trial contract unrelated to the present study. Clinico San Carlos HRI and Curevac have a clinical trial contract unrelated to the present study. JA: Consulting fees from EMA, AEMPS, Almirall, Zeltia; payment for educational events on vaccines from Gilead, Haelix Therapeutics, Merck Sharp & Dohme, Janssen. All other authors declare no competing interests.

## Appendix Supplementary materials

Image, application 1

## References

[1] European Centre for Disease Prevention and Control (ECDC), an agency of the European Union. SARS-CoV-2 variants of concern as of 27 January 2022. https://www.ecdc.europa.eu/en/covid-19/variants-concern. Accessed 31 January 2022.

[2] Ramesh S, Govindarajulu M, Parise RS (2021). Emerging SARS-CoV-2 variants: a review of its mutations, its implications and vaccine efficacy. Vaccines (Basel).

[3] European Centre for Disease Prevention and Control (ECDC). COVID-19 Vaccine Tracker.https://vaccinetracker.ecdc.europa.eu/public/extensions/COVID-19/vaccine-tracker.html#uptake-tab. Accessed 12 December 2021.

[4] European Medicines Agency (EMA). Comirnaty and Spikevax: EMA recommendations on extra doses and boosters. 2021. https://www.ema.europa.eu/en/news/comirnaty-spikevax-ema-recommendations-extra-doses-boosters. Accessed 15 November 2021.

[5] Chung JY, Thone MN, Kwon YJ. (2021). COVID-19 vaccines: the status and perspectives in delivery points of view. Adv Drug Deliv Rev.

[6] Borobia AM, Carcas AJ, Pérez-Olmeda M (2021). Immunogenicity and reactogenicity of BNT162b2 booster in ChAdOx1-S-primed participants (CombiVacS): a multicentre, open-label, randomised, controlled, phase 2 trial. Lancet.

[7] Liu X, Shaw RH, Stuart ASV (2021). Safety and immunogenicity of heterologous versus homologous prime-boost schedules with an adenoviral vectored and mRNA COVID-19 vaccine (Com-COV): a single-blind, randomised, non-inferiority trial. Lancet.

[8] Barda N, Dagan N, Cohen C (2021). Effectiveness of a third dose of the BNT162b2 mRNA COVID-19 vaccine for preventing severe outcomes in Israel: an observational study. Lancet.

[9] Kumar D, Ferreira VH, Hall VG (2021). Neutralization of SARS-CoV-2 variants in transplant recipients after two and three doses of mRNA-1273 vaccine: secondary analysis of a randomized trial. Ann Intern Med.

[10] Kremsner PG, Mann P, Kroidl A (2021). Safety and immunogenicity of an mRNA-lipid nanoparticle vaccine candidate against SARS-CoV-2: a phase 1 randomized clinical trial. Wien Klin Wochenschr.

[11] Kremsner PG, Guerrero RAA, Arana-Arri E (2022). Efficacy and safety of the CVnCoV SARS-CoV-2 mRNA vaccine candidate in ten countries in Europe and Latin America (HERALD): a randomised, observer-blinded, placebo-controlled, phase 2b/3 trial. Lancet Infect Dis.

[12] Stuart LM. (2021). In gratitude for mRNA vaccines. N Engl J Med.

[13] Goel RR, Painter MM, Apostolidis SA (2021). mRNA vaccines induce durable immune memory to SARS-CoV-2 and variants of concern. Science.

[14] Grifoni A, Weiskopf D, Ramirez SI (2020). Targets of T cell responses to SARS-CoV-2 coronavirus in humans with COVID-19 disease and unexposed individuals. Cell.

[15] Bar-On YM, Goldberg Y, Mandel M (2021). Protection of BNT162b2 vaccine booster against Covid-19 in Israel. N Engl J Med.

[16] Kamar N, Abravanel F, Marion O, Couat C, Izopet J, Del Bello A (2021). Three doses of an mRNA Covid-19 vaccine in solid-organ transplant recipients. N Engl J Med.

[17] Hillus D, Schwarz T, Tober-Lau P (2021). Safety, reactogenicity, and immunogenicity of homologous and heterologous prime-boost immunisation with ChAdOx1 nCoV-19 and BNT162b2: a prospective cohort study. Lancet Respir Med.

[18] Munro APS, Janani L, Cornelius V (2021). Safety and immunogenicity of seven COVID-19 vaccines as a third dose (booster) following two doses of ChAdOx1 nCov-19 or BNT162b2 in the UK (COV-BOOST): a blinded, multicentre, randomised, controlled, phase 2 trial. Lancet.

[19] Nordström P, Ballin M, Nordström A. (2021). Effectiveness of heterologous ChAdOx1 nCoV-19 and mRNA prime-boost vaccination against symptomatic Covid-19 infection in Sweden: a nationwide cohort study. Lancet Reg Health Eur.

[20] Dejnirattisai W, Zhou D, Supasa P (2021). Antibody evasion by the P.1 strain of SARS-CoV-2. Cell.

[21] Uriu K, Kimura I, Shirakawa K (2021). Neutralization of the SARS-CoV-2 Mu variant by convalescent and vaccine serum. N Engl J Med.

[22] Gilbert PB, Montefiori DC, McDermott AB (2022). Immune correlates analysis of the mRNA-1273 COVID-19 vaccine efficacy clinical trial. Science.

[23] Cho A, Muecksch F, Schaefer-Babajew D (2021). Anti-SARS-CoV-2 receptor-binding domain antibody evolution after mRNA vaccination. Nature.

