# Supplementary material for: Immune response and reactogenicity after immunization with two-doses of an experimental COVID-19 vaccine (CVnCOV) followed by a third-fourth shot with a standard mRNA vaccine (BNT162b2): RescueVacs multicenter cohort study
Source: eClinicalMedicine. 2022 Jul 1;51:101542. doi: 10.1016/j.eclinm.2022.101542 (PMC9249303; doi:10.1016/j.eclinm.2022.101542)
Supplement: Supplementary file 1 [file mmc1.pdf]

SUPPLEMENTARY APPENDIX 1

Supplementary tables and figures 2
Table S1. SARS-CoV-2 vaccines administered by group 2
Table S2. Baseline characteristics (all vaccinated participants)........ 3
Table S3. Summary statistics for Log-transformed immune response (anti RBD-antibodies measured by ECLIA and neutralizing antibodies determined by pseudovirus assay) by treatment group (modified intention-to-treat population) 4
Figure S1. Evolution of anti RBD-antibodies (ECLIA) and neutralizing titres against reference G614 variant (a) and variants of concern Delta (B.1.617.2), Beta (B.1.351), Mu (B.1.1.529) and Omicron (B.1.1.529) (b), by study group. 5
Table S4. Summary statistics for SARS-CoV-2 antibodies response by seropositivity at baseline: from baseline to visit 2 and from visit 1 to visit 3. Wilcoxon rank-sum (Mann-Whitney) test 6
Table S5. Antibody levels at each visit in subjects who performed visit 3 in 2-5 weeks after the boost dose of BNT162b2. 7
Table S6. Changes in anti-RBD antibodies and NT50 response between visits (intra-group) and study groups (inter-group) in subjects who performed visit 3 in 2-5 weeks after the boost dose of BNT162b2 . 8
Table S7. Evolution of neutralizing antibodies titres (GMT) against reference G614 variant and variants of concern Delta, Beta, Mu and Omicron in patients immunized with two doses of BNT162b2 alone (control) or previously immunized with two doses of CVnCoV (test). 10
Figure S2. Radial graph of reactogenicity (frequency of a) adverse events and b) subjects with adverse events) within 10 days after each BTN162b2 dose by study group. 11
Table S8. Safety: adverse events from day 11 after each BTN162b2 dose onwards. 12
Supplementary Author List 13
RescueVac Study Group 13
Study Protocol 14

Supplementary tables and figures

Table S1. SARS-CoV-2 vaccines administered by group

	n	**Control group (n= 38)**	n	**Test group (n= 92)**
First regimen (first-second dose)	30	**BNT162b2** (Comirnaty, Pfizer-BioNTech; *30 µg/dose*)	92	**CVnCoV** (CureVac, *12 µg/dose*)
	8	**mRNA-1273*** **(Spikevax, Moderna; 100 µg/dose)**		
Booster regimen (third-fourth dose)		-	92	**BNT162b2** (Comirnaty, Pfizer-BioNTech; *30 µg/dose*)

*Excluded from immunogenicity analysis

Table S2. Baseline characteristics (all vaccinated participants)

	Control group (n=38)	Test group (n= 92)	Overall (n=130)
**Age**			
Mean (SD)	29 (9·3)	32 (12·0)	31 (11·3)
Median (IQR)	27 (25–30)	27 (23–45)	27 (23–39)
**Sex, n (%)**			
Female	16 (42%)	36 (39%)	52 (40%)
Male	22 (58%)	56 (61%)	78 (60%)
**Interval between CVnCoV (2nd dose) and BNT162b2 (3rd dose) (days)**			
Mean (SD)	NA	110 (15·4)	110 (15·4)
Median (IQR)	NA	113 (99-122)	113 (99-122)
**Interval between BNT162b2 doses (days)**			
Mean (SD)	23 (3·4)	21 (2·3)	22 (2·7)
Median (IQR)	21 (21-21)	21 (21-21)	21 (21-21)
**Seropositivity, n (%)**			
Anti-RBD titres	0 (0)	89 (98·9%)	89 (68·5%)
Neutralising titres	0 (0)	28 (31·1%)	28 (21·5%)
**Confirmed COVID-19, n (%)**			
>6 months before inclusion	2 (5·3%)	0 (0%)	2 (1·5%)
Peri-vaccination	0 (0)	2 (2·2%)	2 (1·5%)
**Concomitant immunomodulators, n (%)**			
Topical tacrolimus/corticosteroids	0 (0)	1 (1·1%)	1 (0·8%)
Other vaccines (HAV, HPV, Ty21a)	0 (0)	2 (2·2%)	2 (1·5%)
**Comorbidities, n (%)**			
Cancer	0 (0)	0 (0)	0 (0)
Immune deficiencies	0 (0)	0 (0)	0 (0)
Solid organ/stem cell transplant	0 (0)	0 (0)	0 (0)
Chronic kidney disease	0 (0)	0 (0)	0 (0)
Liver disease	0 (0)	0 (0)	0 (0)
Chronic pulmonary disease	0 (0)	0 (0)	0 (0)
Asthma	1 (2·6%)	4 (4·3%)	5 (3·8%)
Hypertension	2 (5·2%)	2 (2·2%)	4 (3·1%)
Heart disease	0 (0)	2 (2·2%)	2 (1·5%)
Diabetes	1 (2·6%)	0 (0)	1 (0·8%)
Neurological disease	0 (0)	0 (0)	0 (0)
Depression	1 (2·6%)	3 (3·3%)	4 (3·1%)
Thalassaemia	0 (0)	0 (0)	0 (0)
Atopic dermatitis/allergic disease	1 (2·6%)	4 (4·3%)	5 (3·8%)
Pregnancy	0 (0)	0 (0)	0 (0)

Table S3. Summary statistics for Log-transformed immune response (anti RBD-antibodies measured by ECLIA and neutralizing antibodies determined by pseudovirus assay) by treatment group (modified intention-to-treat population)

	Control group (n=27) (BNT162b2)			Test group (n= 92) (CVnCoV & BNT162b2)			
	n	GMT	(95% CI)	n	GMT	(95% CI)	GMR (95% CI)
**SARS-CoV-2 anti-RBD (BAU/mL)**							
- Baseline	27	0·4	(0·4-0·4)	92	39·4	(25·6-60·7)	98·6 (44·4-218·8)
- Visit 2	26	29·77	(19·0-46·7)	90	7199·0	(6189·0-8373·8)	241·8 (168·1-347·8)
- Visit 3	22	1832·76	(1262·6-2660·4)	86	11181·0	(10658·2-11729·5)	6·1 (5·0-7·5)
**NT50 _ G614**							
- Baseline	27	16·0	(16·0-16·0)	92	33·2	(24·9-44·3)	2·1 (1·2-3·5)
- Visit 2	26	65·6	(41·4-103·9)	90	2659·8	(2204·7-3209·0)	40·5 (26·6-61·8)
- Visit 3	22	956·4	(712·2-1284·4)	86	2990·5	(2471·8-3618·0)	3·1 (2·1-4·6)
**NT50 _ Delta**							
- Baseline	27	16·0	(16·0-16·0)	92	27·0	20·9-34·8)	1·7 (1·1 - 2·7)
- Visit 2	26	59·6	(32·1-110·7)	90	1813·2	(1422·1-2312·0)	30·4 (17·5 - 52·8)
- Visit 3	22	517·4	(333·8-802·0)	86	2276·9	(1827·0-2837·5)	4·4 (2·7 - 7·1)
**NT50 _ Beta**							
- Baseline	27	16·0	(16·0-16·0)	92	21·7	(17·8-26·5)	1·4 (0·9-2·0)
- Visit 2	26	20·4	(16·0-26·0)	90	926·0	(738·4-1161·3)	45·4 (29·2-70·4)
- Visit 3	22	116·5	(72·7-186·5)	86	1158·8	(950·1-1413·3)	9·9 (6·4-15·8)
**NT50 _ Mu**							
- Baseline	27	16·0	(16·0-16·0)	92	22·1	(18·2-26·8)	1·4 (1·0-2·0)
- Visit 2	26	21·3	(16·5-27·4)	90	1042·6	(832·7-1305·3)	48·9 (31·7-76·0)
- Visit 3	22	175·4	(113·1-271·8)	86	1268·3	(1035·5-1553·5)	7·2 (4·6-11·3)
**NT50 _ Omicron**							
- Baseline	27	16·0	(16·0-16·0)	92	19·9	(17·0-23·3)	1·2 (0·9-1·7)
- Visit 2	26	16·89	(15·10-18·89)	90	493·51	(382·10-637·41)	29·2 (17·9-47·6)
- Visit 3	22	56·99	(35·27-92·10)	86	631·24	(496·92-801·87)	11·1 (6·6-18·6)

GMT: geometric mean titres. GMR: geometric mean ratio. NT50: 50% neutralising antibody titres

Figure S1. Evolution of anti RBD-antibodies (ECLIA) and neutralizing titres against reference G614 variant (a) and variants of concern Delta (B.1.617.2), Beta (B.1.351), Mu (B.1.1.529) and Omicron (B.1.1.529) (b), by study group.

a)

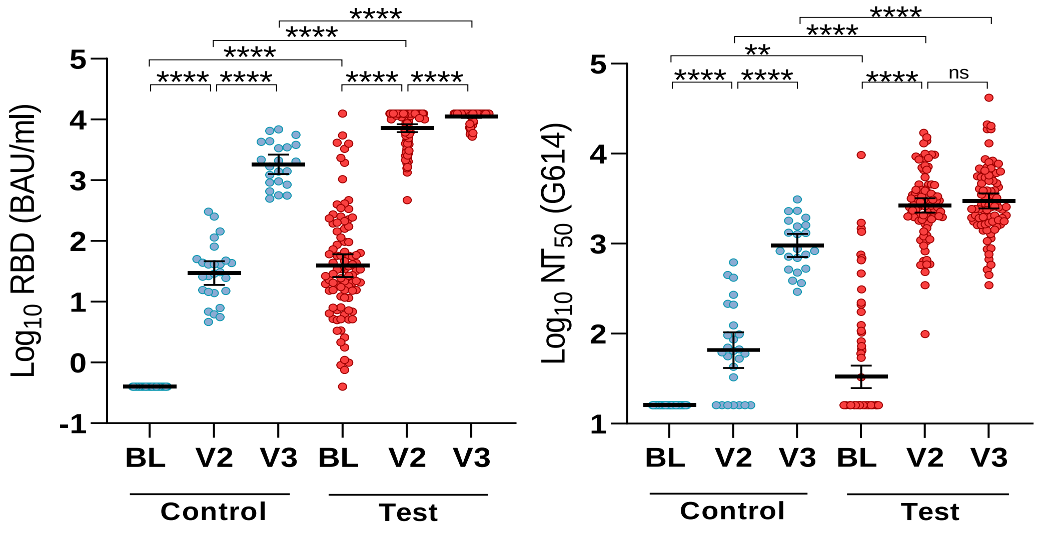

b)

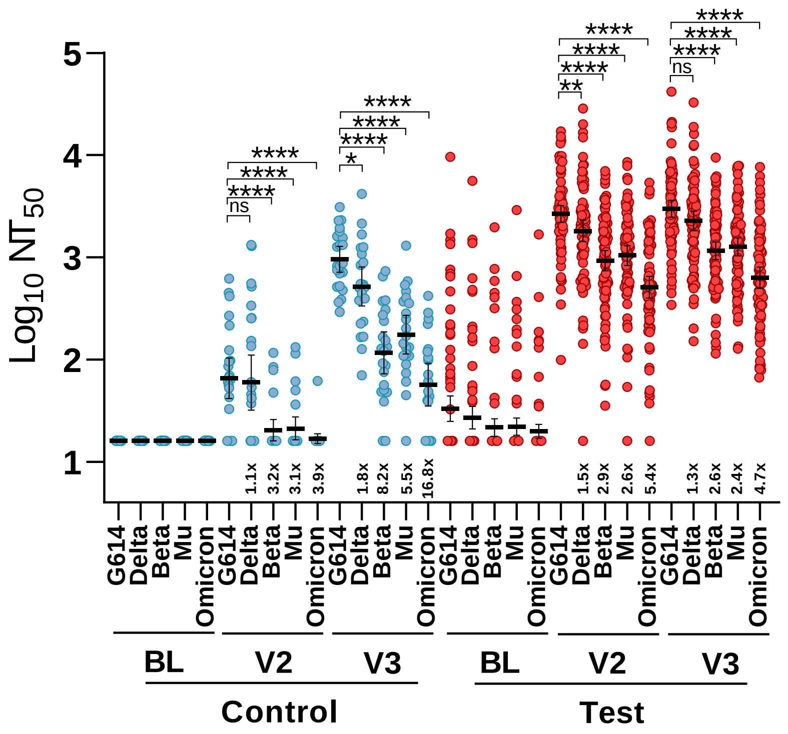

BL: baseline (before first dose of BTN162b2). V2: 3 weeks after fist BTN162b2 dose. V3: 4 weeks after second dose of BTN162b2. Bars indicate geometric mean concentration in each group with a 95% confidence interval. *p <0.05; **p <0.005; ****p <0.0001 using unpaired two-tailed nonparametric Mann-Whitney test; ns: non-significant.

Table S4. Summary statistics for SARS-CoV-2 antibodies response by seropositivity at baseline: from baseline to visit 2 and from visit 1 to visit 3. Wilcoxon rank-sum (Mann-Whitney) test

	**V2–V1** (BNT162b2)			**V3 -V1** (CVnCoV & BNT162b2)		
	z	*p*[1]	*p*[2]	z	*p*[1]	*p*[2]
Anti-RBD	-7·601	<0·00001	0·016	-7·058	<0·00001	0·019
NT50 _G614	-7·622	<0·00001	0·015	-5·227	<0·00001	0·144
NT50 _Delta	-7·182	<0·00001	0·043	-5·264	<0·00001	0·141
NT50 _Beta	-7·640	<0·00001	0·015	-6·562	<0·00001	0·053
NT50 _Mu	-7·824	<0·00001	0·003	-6·262	<0·00001	0·073
NT50 _Omicron	-7·403	<0·00001	0·025	-6·286	<0·00001	0·072

[1]H_1: Seronegative ≠ Seropositive
[2]H1: Seronegative > Seropositive

Table S5. Antibody levels at each visit in subjects who performed visit 3 in 2-5 weeks after the boost dose of BNT162b2.

	Control group (n=19) (BNT162b2)			Test group (n= 56) (CVnCoV & BNT162b2)		
	n	Mean (SD)	Median (IQR)	n	Mean (SD)	Median (IQR)
**SARS-CoV-2 anti-RBD (BAU/mL)**						
- Baseline	19	0·4 (0)	0·4 (0·4-0·4)	56	638·1 (1963·7)	56·1 (18·3-203·0)
- Visit 2	19	71·3 (81·1)	41·3 (26·4-81·2)	56	10571·7 (3120·6)	12500 (9401·0-12500)
- Visit 3	19	2636·1 (2059·6)	2033·0 (915·4-4309·0)	56	11743·8 (1813·7)	12500 (12500-12500)
**NT50 _ G614**						
- Baseline	19	16·0 (0)	16·0 (16·0-16·0)	56	300·1 (1312·7)	16·0 (16·0-66·1)
- Visit 2	19	146·7 (174·9)	64·2 (16·0-214·9)	56	4222·1 (3931·1)	2984·0 (1953·0-4548·0)
- Visit 3	19	1246·7 (766·3)	873·3 (694·0-1795·0)	56	5456·1 (6899·2)	3222·5 (1969·0-5735·0)
**NT50 _ Delta**						
- Baseline	19	16·0 (0)	16·0 (16·0-16·0)	56	177·8 (775·9)	16·0 (16·0-16·0)
- Visit 2	19	219·5 (406·0)	16·0 (16·0-254·9)	56	3494·7 (5185·2)	2004·0 (853·9-3062·5)
- Visit 3	19	852·6 (971·3)	508·6 (235·5-1081·0)	56	4487·4 (5499·8)	2744·0 (1619·5-5475·5)
**NT50 _ Beta**						
- Baseline	19	16·0 (0)	16·0 (16·0-16·0)	56	87·3 (287·5)	16·0 (16·0-16·0)
- Visit 2	19	28·2 (29·7)	16·0 (16·0-16·0)	56	1462·3 (1601·6)	920·8 (419·9-1803·0)
- Visit 3	19	201·0 (205·1)	127·5 (50·3-306·4)	56	1985·6 (1857·3)	1330·5 (757·1-2682·5)
**NT50 _ Mu**						
- Baseline	19	16·0 (0)	16·0 (16·0-16·0)	56	91·6 (388·7)	16·0 (16·0-16·0)
- Visit 2	19	30·1 (34·0)	16·0 (16·0-16·0)	56	1523·9 (1690·6)	(526·3-2018·0)
- Visit 3	19	285·7 (297·1)	170·9 (108·7-401·7)	56	2166·5 (1964·3)	(825·8-2731·5)
**NT50 _ Omicron**						
- Baseline	19	16·0 (0)	16·0 (16·0-16·0)	56	54·5 (223·1)	16·0 (16·0-16·0)
- Visit 2	19	18·4 (10·5)	16·0 (16·0-16·0)	56	909·0 (1133·0)	457·6 (200·4-1284)
- Visit 3	19	105·2 (112·2)	60·9 (16-126·7)	56	1363·9 (1629·3)	801·4 (324·1-1605·5)

Table S6. Changes in anti-RBD antibodies and NT50 response between visits (intra-group) and study groups (inter-group) in subjects who performed visit 3 in 2-5 weeks after the boost dose of BNT162b2

	**Control group (n=19) (BNT162b2)**				**Test group (n= 56) (CVnCoV & BNT162b2)**				**Inter-group comparisons**		
	n	Absolute intragroup change			n	Absolute intragroup change					
		Mean (SD)	Median (IQR)	ZW		Mean (SD)	Median (IQR)	ZW	WW	Z	p-value
**SARS-CoV-2 anti-RBD (BAU/mL)**											
Visit 2 - baseline	19	70·9 (81·1)	40·9 (26·0-80·8)	3·82 (p<0·00001)	56	≥9933·5 (3373·3)	12236·7 (≥7976·2-12443·0)	6·51 (p<0·00001)	209	-6·25	<0·00001
Visit 3 - visit 1	19	2635·7 (2059·5)	2032·6 (915·0-4308·6)	3·82 (p<0·00001)	56	≥11105·6 (2487·1)	12348·6 (≥10869·4-12459·1)	6·51 (p<0·00001)	214	-6·19	<0·00001
Visit 3 - visit 2	19	2564·7 (2007·0)	1991·7 (890·6-4195·4)	3·8 (p<0·00001)	56	≥1172·1 (2082·1)	0 (≥0-2423)	4·09 (p<0·00001)	1028	3·95	<0·0001
**NT50 _ G614**											
Visit 2 - baseline	19	130·7 (174·9)	48·2 (0-198·9)	3·56 (p=0·0005)	56	3922·1 (4030·3)	2829·5 (1443·1-4518·0)	6·15 (p<0·0001)	216	-6·17	<0·00001
Visit 3 - visit 1	19	1230·7 (766·3)	857·3 (678·0-1779·0)	3·82 (p<0·00001)	56	5156·0 (7048·9)	2684·0 (1923·0-5719·0)	6·14 (p<0·00001)	338	-4·68	<0·00001
Visit 3 - visit 2	19	2000·0 (702·7)	811·4 (499·2-1525·6)	3·82 (p<0·00001)	56	1233·9 (6188·4)	347·0 (-570·5 - 1961·50)	1·77 (p=0·0776)	877	1·89	0·0594
**NT50 _ Delta**											
Visit 2 - baseline	19	203·5 (406·0)	0 (0-238·9)	2·96 (p=0·0039)	56	3316·8 (5145·1)	1933·0 (667·0-2953·5)	6·51 (p<0·00001)	254	-5·71	<0·00001
Visit 3 - visit 1	19	836·6 (971·3)	492·6 (219·5-1065·0)	3·82 (p<0·00001)	56	4309·6 (5505·0)	2590·5 (1476·1-4652·0)	6·51 (p<0·00001)	333	-4·74	<0·00001
Visit 3 - visit 2	19	633·1 (968·2)	370·8 (166·8-510·6)	3·62 (p<0·0001)	56	992·7 (5911·2)	384·7 (-56·7-1105·0)	2·77 (p=0·0051)	726	0·05	0·9663
**NT50 _ Beta**											
Visit 2 - baseline	19	12·2 (29·7)	0 (0-0)	1·73 (p=0·2500)	56	1375·0 (1577·8)	839·9 (379·5-1776·3)	6·27 (p<0·00001)	215	-6·21	<0·00001
Visit 3 - visit 1	19	185·0 (205·2)	111·5 (34·3-290·4)	3·77 (p<0·00001)	56	1898·3 (1871·4)	1314·5 (702·5-2515·3)	6·31 (p<0·00001)	245	-5·81	<0·00001
Visit 3 - visit 2	19	172·8 (205·2)	111·5 (32·6-290·4)	3·77 (p<0·00001)	56	523·3 (1553·2)	302·0 (-81·6-753·5)	3·00 (p=0·0023)	652	-0·85	0·4003

**NT50 _ Mu**										
- Visit2 - baseline	19	14·1 (34·0)	0 (0-0)	2·00 (p=0·1250)	56	1432·3 (1647·5)	1044·5 (501·7-1814·6)	6·51 (p<0·00001)	197	-6·42	<0·00001
- Visit 3 - visit 1	19	269·7 (297·1)	154·9 (92·7-385·7)	3·81 (p<0·00001)	56	2074·9 (1984·3)	1511·0 (751·8-2539·8)	6·39 (p<0·00001)	271	-5·49	<0·00001
- Visit 3 - visit 2	19	255·6 (298·6)	154·9 (73·6-385·7)	3·81 (p<0·00001)	56	642·6 (1203·1)	295·6 (26·1-1126·0)	4·50 (p<0·00001)	633	-1·08	0·2836
**NT50 _ Omicron**										
- Visit2 - baseline	19	2·4 (10·5)	0 (0-0)	1·00 (p=1·0000)	56	854·4 (1125·7)	433·2 (175·6-1175·0)	6·33 (p<0·00001)	233	-6·01	<0·00001
- Visit 3 - visit 1	19	89·2 (112·2)	44·8 (0-110·7)	3·56 (p<0·0001)	56	1309·4 (1639·2)	719·2 (263·8-1589·5)	6·31 (p<0·00001)	286	-5·31	<0·00001
- Visit 3 - visit 2	19	86·8 (112·7)	42·5 (0-110·7)	3·56 (p<0·0001)	56	455·0 (940·1)	176·5 (12·5-584·1)	4·15 (p<0·00001)	577	-1·77	0·0782

p-values obtained using unpaired two-tailed nonparametric Mann-Whitney test

Table S7. Evolution of neutralizing antibodies titres (GMT) against reference G614 variant and variants of concern Delta, Beta, Mu and Omicron in patients immunized with two doses of BNT162b2 alone (control) or previously immunized with two doses of CVnCoV (test).

	Control group (n=27) (BNT162b2)			Test group (n= 92) (CVnCoV & BNT162b2)			p-value
	n	GMT (95%CI)	fold	n	GMT (95%CI)	fold	
**Baseline**							
G614	27	16·0 (16·0-16·0)	1·0	92	33·2 (24·9-44·3)	1·0	0·0027
Delta	27	16·0 (16·0-16·0)	1·0	92	27·0 (20·9-34·8)	1·2	0·0153
Beta	27	16·0 (16·0-16·0)	1·0	92	21·7 (17·8-26·5)	1·5	0·114
Mu	27	16·0 (16·0-16·0)	1·0	92	22·1 (18·2-26·8)	1·5	0·0658
Omicron	27	16·0 (16·0-16·0)	1·0	92	19·9 (17·0-23·3)	1·7	0·1301
**Visit 2**							
G614	26	65·6 (41·4-103·9)	1·0	90	2659·8 (2204·7-3209·0)	1·0	<0·0001
Delta	26	59·6 (32·1-110·7)	1·1	90	1813·2 (1422·1-2312·0)	1·5	<0·0001
Beta	26	20·4 (16·0-26·0)	3·2	90	926·0 (738·4-1161·3)	2·9	<0·0001
Mu	26	21·3 (16·5-27·4)	3·1	90	1042·6 (832·7-1305·3)	2·6	<0·0001
Omicron	26	16·9 (15·1-18·9)	3·9	90	493·5 (382·1-637·4)	5·4	<0·0001
**Visit 3**							
G614	22	956·4 (712·2-1284·4)	1·0	86	2990·5 (2471·8-3618·0)	1·0	<0·0001
Delta	22	517·4 (333·8-802·0)	1·8	86	2276·9 (1827·0-2837·5)	1·3	<0·0001
Beta	22	116·5 (72·7-186·5)	8·2	86	1158·8 (950·1-1413·3)	2·6	<0·0001
Mu	22	175·4 (113·1-271·8)	5·5	86	1268·3 (1035·5-1553·5)	2·4	<0·0001
Omicron	22	57·0 (35·3-92·1)	16·8	86	631·2 (496·9-801·9)	4·7	<0·0001

Baseline: before first BNT162b2 dose. Visit 2: before second dose of BNT162b2 dose. Visit 3: 28 days after second BNT161b2 dose. Fold values show decrease in sensitivity to different variants as compared with G614 at different times and group of patients; *p*-values obtained using unpaired two-tailed nonparametric Mann-Whitney test.

Figure S2. Radial graph of reactogenicity (frequency of a) adverse events and b) subjects with adverse events) within 10 days after each BTN162b2 dose by study group.

a)

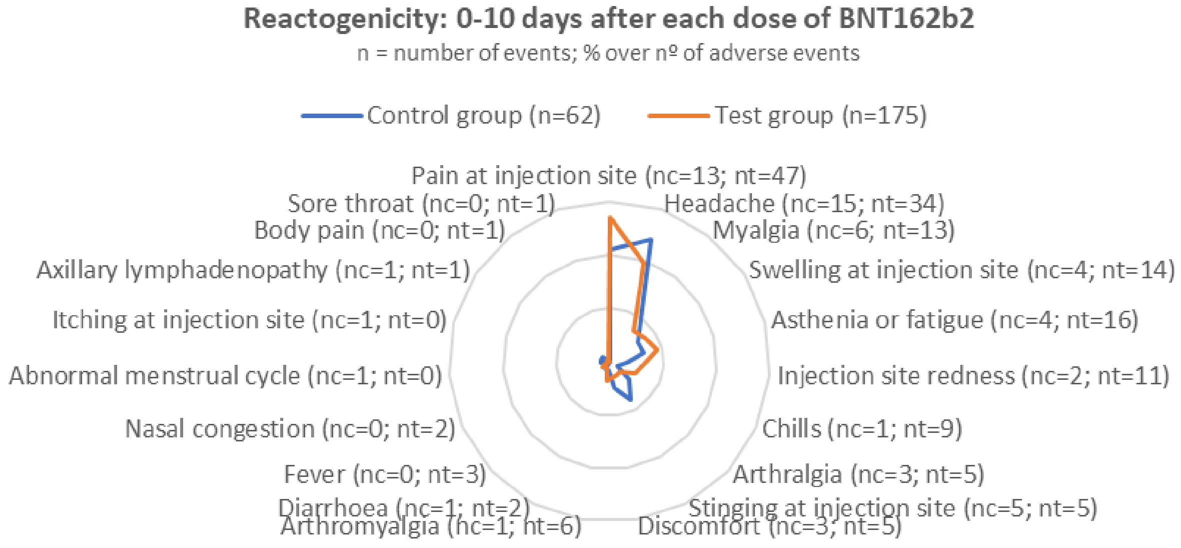

b)

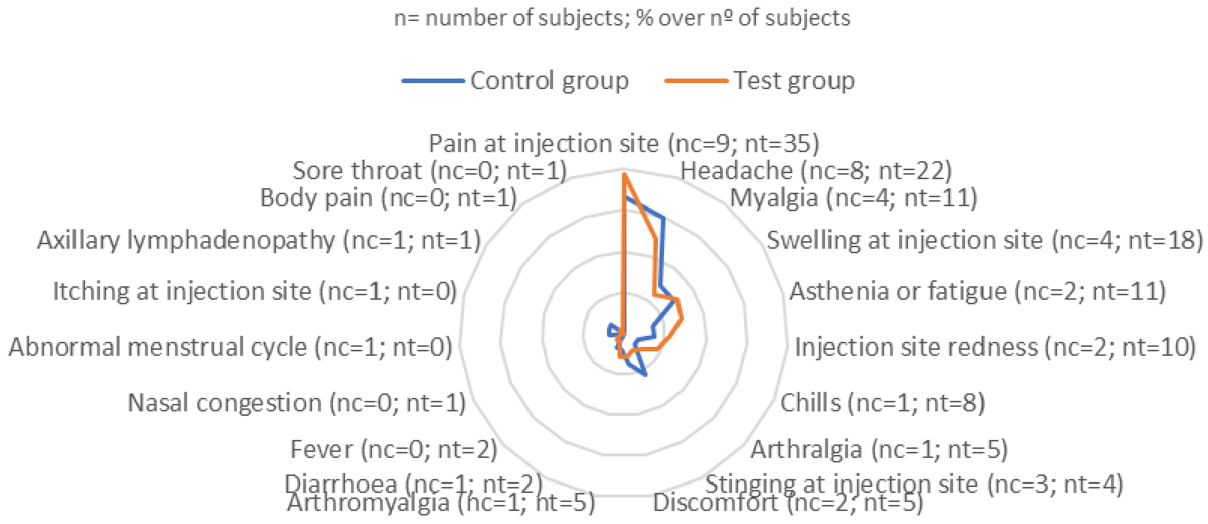

Table S8. Safety: adverse events from day 11 after each BTN162b2 dose onwards.

Adverse event	Group	Days after prime BNT162b2	Intensity	SAE
Discomfort	CONTROL	12	Mild	No
Headache	CONTROL	13	Mild	No
Abnormal menstrual cycle	CONTROL	.	Mild	No
Sore throat	TEST	11	Mild	No
Pruritus ani	TEST	12	Mild	No
Headache	TEST	13	Mild	No
Headache	TEST	13	Mild	No
Headache	TEST	16	Mild	No
Headache	TEST	18	Mild	No
Fracture _hand	TEST	34	Moderate	No
Fracture _toe	TEST	36	Mild	No
Penis injury	TEST	47	Moderate	No
Abdominal pain	TEST	48	Mild	No
Constipation	TEST	48	Mild	No
Low-grade fever	TEST	51	Mild	No
Cold	TEST	51	Mild	No
Headache	TEST	51	Mild	No
Dermatitis exacerbation_Hands dyshidrosis	TEST	61	Moderate	No
Retained Cu-IUD	TEST	61	Moderate	No
Asthenia	TEST	65	Moderate	No
Tonsillitis	TEST	71	Moderate	No
Dermatomycosis	TEST	83	Mild	No
Pregnancy	TEST	89	Moderate	No
Dermatitis	TEST	99	Mild	No
Cold	TEST	109	Mild	No

Supplementary Author List

RescueVac Study Group

AFFILIATION	SURNAME	NAME
**Hospital Clínico San Carlos-IdISSC. Madrid, Spain**	Rodríguez Galán	Natalia
	Zhu Huang	Ouhao
	González Rojano	Esperanza
	Lozano Martín	Daniel
	Rivas-Paterna	Ana Belén
	Delgado-Iribarren	Alberto
	Fuentes Ferrer	Manuel
	Sánchez del Hoyo	Rafael
	Mato Chain	Gloria
	García Lavandeira	Celia
	Gómez Mayoral	Beatriz
	Hermoso Núñez de Arenas	Elena
**IIS Biocruces Bizkaia. Barakaldo, Spain**	Gallego	Mikel
	Lazaro	Maria Angeles
	García-Vazquez	María Dolores
	Solaun	Miren Sorne
	Cobos-Fraile	Susana
	Osorio	Maria Eugenia
	García de Vicuña	Aitor
	Santorcuato	Ana
	de Benito	Sara
	Exposito	Iraide
**Hospital Universitario Donostia - IIS BIODONOSTIA. San Sebastián, Spain**	Etxart Lasa	María Pilar
**IIS BIODONOSTIA. San Sebastián, Spain**	Rico	Leonor
**AIDS Immunopathogenesis Unit. Instituto de Salud Carlos III. Madrid, Spain**	Cascajero	Almudena
	Jiménez-Santana	Paloma
	Calonge	Esther
**Centro Nacional de Microbiología. Instituto de Salud Carlos III. Madrid, Spain**	Fedele<br>Perea	Giovanni<br>Concepción

Study Protocol

Protocol Code: RescueVacs

Immunogenicity response study of licensed COVID-19 vaccines in subjects who have previously received an experimental vaccine.

Version: 1.0

Version Date: 14/07/2021

Principal Investigators:

Dr. Antonio Portolés. Clinical Pharmacology Department. Clinico San Carlos Hospital C/ Prof. Martín Lagos s/n 28040 – MADRID (Spain). Phone Number: +34 913303413
Dr. Eunate Arana. University Hospital Cruces. C/Cruces Plaza, S/N, 48903 Barakaldo, Bizkaia (Spain).
Dr. Itziar Vergara. Donostia University Hospital. C/Begiristain Doktorea Pasealekua, s/n, 20014 Donostia, Gipuzkoa (Spain).

Co-Investigators:

Clínico San Carlos Hospital:
Oliver Astasio, Ana Ascaso, Daniel Lozano, Ana Belén Rivas, Carla Pérez Ingidua, Natalia Rodríguez, Elena Hermoso, Emilio Vargas, Alberto Mariano Lázaro, Gloria Mato Chain, Beatriz Gómez Mayoral, Alberto Delgado
HIV Immunopathology Lab & CNM (ISCIII)
Spanish National Centre for Microbiology as a participanting centre for analysis.
Mayte Pérez Olmeda, Javier García-Pérez, José Alcamí

Ethics Committee for Clinical Investigation

The approval of the CEIm of the Clínico San Carlos Hospital will be requested.

1. BACKGROUND AND RATIONALE FOR THE STUDY 4
2. HIPOTHESIS 5
3. OBJECTIVES 5
4. VARIABLES 5
5. METHODS 6
5.1. Design 6
5.2. Population/ Study Sample 6
5.3. Procedures 6
6. STATISTICAL ANALYSIS PLAN 6
7. ETHICAL CONSIDERATIONS 7
7.1. Privacy and confidentiality 7
7.2. Data Quality and Document Custody 7
8. FINANCIAL DISCLOSURE 7
9. BIBLIOGRAPHY 7

1. Background and rationale for the study

Since the beginning of 2020, the world has experienced an unprecedented pandemic caused by the SARS-COV-2 virus, began in Wuhan (China). The arrival of the first licensed vaccines in 2020 has offered hope for a solution.

After solving doubts about mechanisms of action, efficacy and safety, and having several vaccines already on the market, there are still unknowns about the duration of the effects, efficacy against different variants of the virus, and possible incompatibilities and possibilities of mixing and matching vaccines.

The emerging SARS-CoV-2 variants of concern (VoC) (1) have complicated the study of new vaccines and the assurance of the length of the effects of those already marketed. Besides, the excessive demand for the supply of these products, leads us to the need to keep active the research to discover and produce new vaccines.

Regarding to the administration guidelines, doses, number of doses and possible combinations of vaccines in different situations and ages, there are many questions to be resolved. Thus, different studies and guidelines made possible to recommend the use of a single dose of vaccine in patients who have recently passed COVID-19, recommendation which may change depending on the age of the subject (2). Also, some voices alerts about the need to use booster doses in vaccination regimens without forgetting the doubts about the supply complications. Complications which could lead to a combination between the usual two doses ("prime" and "boost") (3,4).
Many other situations such as possible safety problems not previously identified, or the differential effect in previously unidentified subpopulations, poses the question about the need to implement variations respect the treatment previously established.

Two studies have recently been published as first experiences in heterologous vaccination: COMBIVACS and COMCOV (3,4). Both of them, shows that the combination of a first dose of Vaxzevria and a boost with Comirnaty generated robust responses in terms of immunogenicity and an tolerable reactogenicity profile.
In this case, the doubts generated about the safety of the first of these vaccines was taken advantage of.

As time goes and the pandemic progresses and a growing part of the population begins to be vaccinated, the complexity for the study of new vaccines increases and thinking of alternative methods becomes a necessity. Besides, supply reasons, -among others-, lead to the need for explore the efficacy and safety of the possible combinations of different vaccines. Due to these difficulties, the information can be obtained from aleatorized clinical trials but also from specific cases or uses.

Nowadays, there are situations in which a subject must to receive more than one vaccination regimen, actually, could happen someone have to receive a vaccine that has

not been authorized by a regulatory agency. As an example, we can show a case in which participants of a clinical trial, treated on our Spanish centres, have received a vaccine which has not been able to demonstrate a sufficiently high efficacy to allow an accelerated authorization. This population is awaiting to receive a heterologous regimen (rescue regimen) in the context of routine clinical practice, to achieve a complete immunization. Thus, this situation can be used to collect information about the immunogenicity of combinations and repeated vaccinations.

Due to all the doubts that persist about vaccines against COVID-19 and the need to obtain knowledge about them, it is important to seize opportunities that are presented to us to generate it. Therefore, in the presence of a population that is expected to be revaccinated in the context of routine clinical practice, this academic and non-profit study is proposed to study the immunogenicity of the administration of different vaccination regimens.

2. Objectives

Main Objective:
To assess the humoral immune response against SARS-CoV-2 after the respective national vaccination program doses, in subjects who had previously received experimental COVID vaccination, in comparison with those who had not previously received any vaccine.

Secondary Objectives:
To assess the long-term humoral immune response against SARS-CoV-2 (6 months after completing the doses of the vaccination program).

To evaluate the neutralizing antibody levels using viral pseudotypes carrying the SARS-CoV-2 protein S of different variants.

3. Variables

3.1. Main Variable

Serological response to vaccination: antibodies against the receptor-binding domain (anti-RBD) of the S region of SARS-CoV-2 measured by immunoassay, prior to the vaccine doses and 2-5 weeks after the last authorized vaccine dose.

3.2.Secondary Variables

Serological response to vaccination: antibodies 6 months ± 21 days after completing the vaccination.

3.3.Independent Variables

The information about sex, date of birth, experimental vaccine previously received and date of administration, vaccination regimen, commercial vaccine received and date of

administration, previous SARS-CoV-2 infection (confirmed by PCR or antigen test), severity (hospital admission yes/ no) and date of infection, will be used as classification or fit factors.

4. Methodology

4.1. Design

A descriptive, observational study about the immune response and searching for potential response factors will be done. The Study will be conducted in adult subjects (>=18 years) vaccinated against SARS-CoV-2 within the national vaccination program.

Blood samples from subjects vaccinated within the national vaccination program, whether they had been previously vaccinated with an experimental vaccine or had not yet been vaccinated, will be analyze: pre-doses, 2-5 weeks after last dose and +6 months after last dose.

Subjects who had previously received placebo or those who had not participated in any covid vaccine study and therefore had not received any covid vaccine, will be used as control group.

Subjects who attend the vaccination point will be informed about SARS-CoV-2 immunogenicity studies and donations to the Biobank. Those who wish to participate must have understood, accepted and signed the informed consent for the transfer and storage of samples in the Biobank and then for participation in the study.

Under no circumstances their participation may affect their vaccination regimen.

Immunogenicity analyses will be carried out in the Immunology laboratory of the ISCIII Majadahonda.

4.2. Population/Study Sample

Adults (18 years) who should receive an authorized vaccine within the national vaccination program at participating centres.

4.2.1. Inclusion Criteria

Participants must meet all the inclusion criteria:

1. Subjects over 18 years of age who come to receive an authorized prime-boost vaccination regimen against COVID-19 according to the current national vaccination program and accepted to donate blood samples to the respective biobank aimed at the study of antiSARS-CoV-2 immunogenicity.
2. Subjects who give their written consent indicating that they wish to participate in the study and understand the purpose and procedures of the study.
3. Subjects who are willing and able to comply with the study procedures.
4. Clinically stable subjects.

4.2.2. Exclusion Criteria

Any participant who meets any of the following criteria should not be included in the study:

1. Subjects who have already received any already authorized by the national vaccination plan vaccine.
2. Current clinically significant acute diseases (this does not include minor diseases such as diarrhea or mild upper respiratory tract infection), relevant immune diseases, immunosuppresion, systemic biologic inmunotherapy, or situations that in the opinion of the investigator may interfere with the results of the study.
3. Known or suspected allergies to any component of the administered vaccines.

4.2.3. Withdrawal Criteria

1. Withdrawal of informed consent.
2. Any condition that compromises the participant's ability to continue with the study procedures or that involves the loss of ability to freely consent.
3. Participants who meet any other exclusion criteria, disease or condition which could interfere with the results of the study. Also, incidents that, in the opinion of the researcher, violate the convenience of their participation or invalidate their contribution to respond to research questions.
4. Non-compliance with the study procedures at the discretion of the researcher.
5. Due to the closure of the centre or premature termination of the study.
6. According to the request of the AEMPS or the EMA, an Institutional Review Board (IRB) or the Clinical Research Ethics Committee.

In accordance with the provisions of the Declaration of Helsinki (Fortaleza, Brazil, October 2013) and the current legislation, participants may withdraw their consent to participate without having to justify their decision and without prejudice their medical care.

Withdrawal of informed consent means that any new information about the participant will be collected or processed. In addition, any study procedure will be performed.

4.3. Procedures

The informed consent of the patients will be obtained at the hospital vaccination point. Patients who had accepted to donate the following blood samples to the biobank collection aforementioned:
*Baseline sample, prior to the first vaccine dose;
*prior to the second vaccine dose;
*at 2-5 weeks after completing the vaccination regimen;
*6 months after completing the authorized vaccination regimen.

Immunogenicity analyses will be sent and analyse at the immunology laboratory of the ISCIII once the samples will be obtained from biobank.

5. Lab Procedures

Centre's standard venipuncture procedure must guarantee biosafety measures at all times:

Clot activation collection tube (brown cap) should have been used and the sample gently inverted between 8 to 10 times. Then, kept standing upright until processing.

The centrifugation of the samples should be carried out as soon as possible, within 4 hours after extraction at room temperature for 10 minutes at 2000g or for 5 minutes at 3000g.

The serum will be carefully pipetted the into the 4 transport cryovials:

- Cryovial 1: 1 ml.
- Cryovial 2: 1 ml
- Cryovial 3: At least 0.5 ml
- Cryovial 4: At least 0.5 ml

All the cryovials will be stored frozen at -20ºC in a freezer with probe for refrigeration monitoring.

All the samples will be transported in a three-isothermal packaging inside refrigerators (5,6) with dry ice and temperature control to Spanish National Centre for Microbiology (CNM-ISCIII) for their analysis.

6. Statistical Analysis Plan

The analyses will be performed using a validated statistical software, such as R, SPSS or Stata.

A descriptive analysis of demographic and clinical variables, days elapsed between visits and vaccinations, and antibody titers will be performed. Data were summarised using absolute and relative frequencies (based on the non-missing sample size) for categorical variables and mean and SD, or 1st quartile, median, 3rd quartile, maximum and minimum for numerical variables. Minimum and maximum values were also presented for numerical variables. The population will be described by subgroups according to parameters such as their sex, age, treatment group or COVID-19 infection. The normality of continuous variables will be tested.

The number and flowchart of participants will be presented. The screening period and the total number of subjects enrolled will be presented by treatment group. Also, those that were included in the complete analysis set, in the immunogenicity analysis and the safety analysis set will be presented.

Primary immunogenicity endpoints will be analysed to the extent that normality has been achieved with an ANCOVA test adjusted for possible covariates, such as treatment received or SARS-CoV-2 infection.

An inferential analysis will be conducted to identify any significant changes in categorical variables over time and between groups. The primary analysis will compare the anti-spike IgG increase achieved at visits 2 and 3 with respect to baseline and between groups.

Secondary analysis for immunogenicity outcomes will be performed on neutralizing antibodies, differences in titers over the time and days elapsed between doses.

<u>Sample size</u>

It is expected to include at least 30 subjects per center (2/3 who had previously received another experimental regimen, and 1/3 who had not), preferably of different age groups of interest, for the study of immunogenicity and response factors of the COVID vaccines.

7. Ethical Considerations

The study will be carried out under conditions of respect for the fundamental rights of the people, the ethical principles for conduct human research and the treatment of their biological samples. The standards of Good Clinical Practice (CPMP / ICH / 135 / 95 and ICH E6), the ethical principles established in the Declaration of Helsinki (Fortaleza, 2013) and in the Convention for the protection of Human Rights and Biomedicine (Oviedo 1997), ratified in 1999, as well as the Law 14/2007 of 3 of July 2007, on Biomedical Research and 1716/2011 Royal Decree (RD) will be respected. The study will be evaluated by an accredited Clinical Research Ethics Committee (CEIm).

7.1. Privacy and confidentiality

The data collection will be carried out in accordance with the Good Clinical Practice Standards. The biological samples of the participants will come from an accredited biobank and will be pseudonymised. The treatment, communication, and transfer of the data will comply with the provisions of 3/2018 Organic Law, of December 5, on the Protection of Personal Data and guarantee of digital rights.

7.2. Data Quality and Document Custody

Participant data will be obtained from their medical history (source document). All of them will be registered in the eCRF (REDCap platform) to be analyse. The researcher is responsible to confirm that data entered into the eCRF by authorized site personnel are accurate, complete and correct by physically or electronically signing the eCRF.
The investigator will allow study monitoring, audits, review and inspections and will provide direct access to source documents.
The researcher will keep the study records and documents for at least 15 years after its completion, unless local regulations or institutional policies require a longer period.

8. Financial Disclosure

Any financial compensation will be received by the researchers or the hospital for their collaboration. This is an observational study carried out under normal conditions. Each center will carry out the evaluations using its own means. The ISCIII's Immunology laboratory will carry out inmunogenicity response analysis.

9. Bibliography

1. Sanyaolu A, Okorie C, Marinkovic A, Haider N, Abbasi AF, Jaferi U, Prakash S, Balendra V. The emerging SARS-CoV-2 variants of concern. Ther Adv Infect Dis. 2021 Jun 18;8

2.Shaw RH, Stuart A, Greenland M, Liu X, Nguyen Van-Tam JS, Snape MD; Com-COV Study Group. Heterologous prime-boost COVID-19 vaccination: initial reactogenicity data. Lancet. 2021 May 29;397(10289):2043-2046.

3. Immunogenicity and reactogenicity of BNT162b2 booster in ChAdOx1-S-primed participants (CombiVacS): a multicentre, open-label, randomised, controlled, phase 2 trial.

4. Estrategia de Vacunación frente a COVID-19. Ministerio de Sanidad del Gobierno de España. Visitado el 30/06/2021. Disponible: https://www.mscbs.gob.es/profesionales/saludPublica/prevPromocion/vacunaciones/covid19/docs/Actualizacion_EstrategiaVacunacion.

5. Meyer B, Torriani G, Yerly S, Mazza L, Calame A, Arm-Vernez I, Zimmer G, Agoritsas T, Stirnemann J, Spechbach H, Guessous I, Stringhini S, Pugin J, Roux-Lombard P, Fontao L, Siegrist CA, Eckerle I, Vuilleumier N, Kaiser L; Geneva Center for Emerging Viral Diseases. Validation of a commercially available SARS-CoV-2 serological immunoassay. Clin Microbiol Infect. 2020 Oct;26(10):1386-1394.

6. DiaSorin. LIAISON SARS-CoV-2 TrimericS IgG assay. https://www.diasorin.com/sites/default/files/allegati_prodotti/liaisonr_sars-cov-2_trimerics_igg_assay_m0870004408_a_lr.pdf Date accessed: July 14, 2021
